# Inhibition of CD45-specific phosphatase activity restores the differentiation potential of aged mesenchymal stromal cells: implications in regenerative medicine

**DOI:** 10.1186/s40659-025-00603-8

**Published:** 2025-05-02

**Authors:** Madhurima Das, Isha Behere, Ganesh Ingavle, Anuradha Vaidya, Vaijayanti Prakash Kale

**Affiliations:** 1 Symbiosis Centre for Stem Cell Research, Symbiosis International (Deemed University), Pune, 412115 India; 2 Symbiosis School of Biological Sciences, Symbiosis International (Deemed University), Pune, 412115 India; 3 NIHR Biomedical Research Centre, Guy’s & St Thomas’ NHS Foundation Trust and King’s College London Clinical Research Facility, London, UK; 4 Symbiosis Centre for Stem Cell Research (SCSCR), Symbiosis School of Biological Sciences, Symbiosis Knowledge Park, Lavale, Pune, 412112 India

**Keywords:** Mesenchymal stem/stromal cells, CD45, Protein tyrosine phosphatase, Regulatory kinases, Differentiation, Regenerative medicine

## Abstract

**Background:**

Aging affects the reparative potency of mesenchymal stem/stromal cells (MSCs) by diminishing their proliferation and differentiation capability; making them unsuitable for regenerative purposes. Earlier we showed that MSCs acquire the expression of CD45 as a consequence of aging, and this increased expression is associated with downregulated expression of osteogenic markers and upregulated expression of adipogenic and osteoclastogenic markers. However, whether CD45 is actively involved in the aging-mediated deregulated differentiation in the MSCs was not elucidated.

**Results:**

In the present study, we showed that pharmacological inhibition of CD45-specific phosphatase activity in the aged MSCs restores their differentiation potential to young-like. Investigation of the molecular mechanism involved in the process showed that several regulatory kinases like p38, p44/42, Src, and GSK3β are in their dephosphorylated form in the aged MSCs, and importantly, this status gets reversed by the application of a CD45-specific PTP inhibitor. Conversely, pharmacological inhibition of these kinases in young MSCs imposes an aged-like gene expression profile on them. Additionally, we also showed that the secretome of aged MSCs affects the viability and differentiation of primary chondrocytes, and this detrimental effect is reversed by treating aged MSCs with the PTP inhibitor. Our data demonstrate that the aging-mediated expression of CD45 in MSCs alters their differentiation profile by dephosphorylating several kinases and treating the aged MSCs with a CD45 PTP activity inhibitor rejuvenates them.

**Conclusions:**

CD45 can be used as an aging marker for mesenchymal stem cells. Alteration of CD45 phosphatase activity could have significant implications for the use of MSCs in regenerative medicine.

**Graphical abstract:**

The rejuvenating effect of CD45-specific PTP inhibitor on aged MSCs: Aging diminishes the reparative potency of mesenchymal stem/stromal cells (MSCs) by increasing CD45 expression in them. This increased expression of CD45 leads to the downregulation of osteogenic and chondrogenic markers and upregulation of adipogenic and osteoclastogenic markers. Inhibition of CD45-specific phosphatase activity in aged MSCs restores their differentiation potential to young-like by restoring the phosphorylation status of various regulatory kinases (p38, p44/42, Src, and GSK3β). Elevated expression of osteoclastic markers in aged MSCs, also reversed after CD45-specific PTP inhibitor treatment. These findings suggest that targeting CD45 phosphatase activity could enhance the regenerative capabilities of aged MSCs, making them more suitable for therapeutic applications

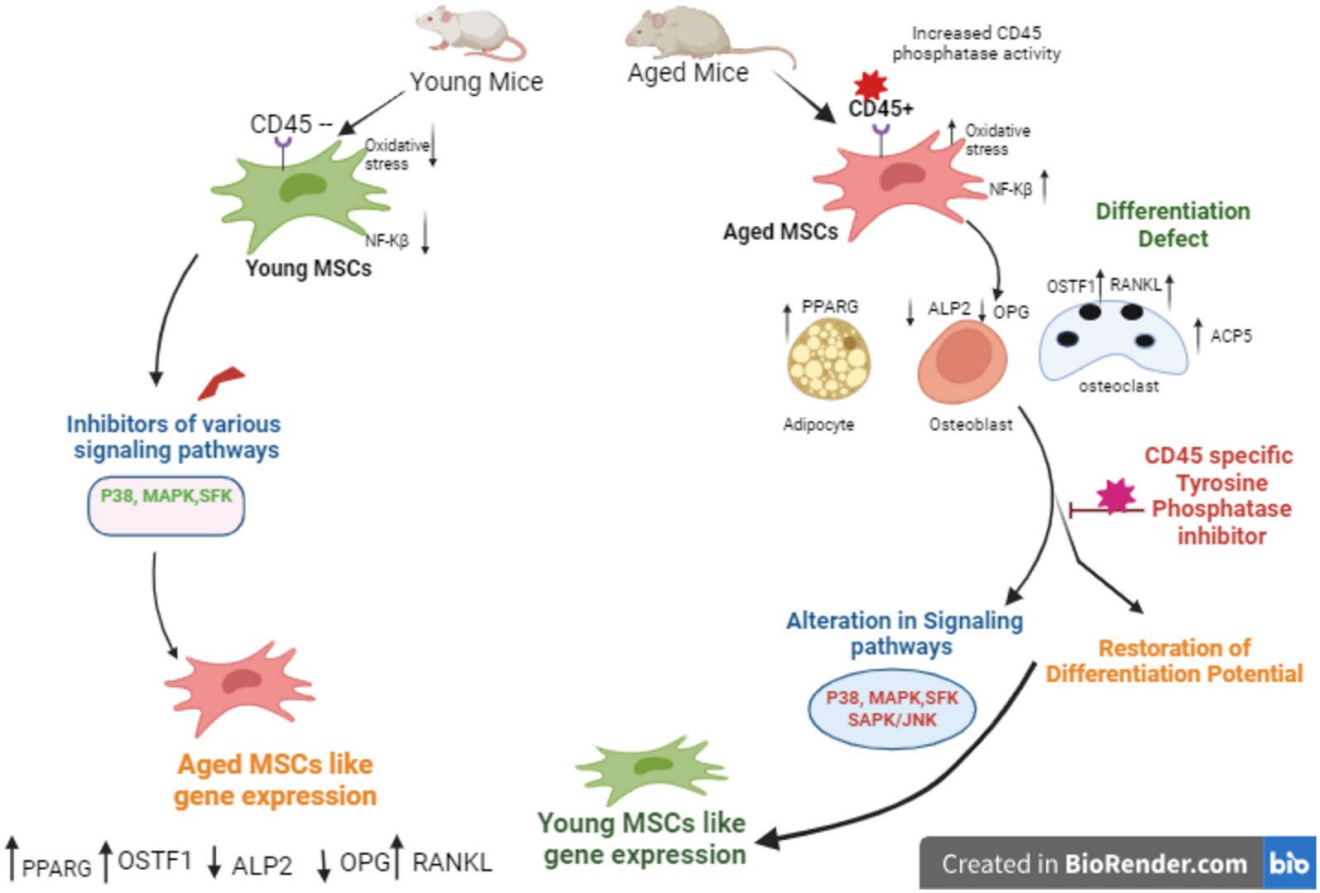

**Supplementary Information:**

The online version contains supplementary material available at 10.1186/s40659-025-00603-8.

## Background

Owing to their multi-lineage differentiation potential, self-renewal capacity, immunoregulatory properties, etc. mesenchymal stem/stromal cells (MSCs) have formed an important option in regenerative medicine protocols [1, 2]. The regenerative potential of MSCs extends beyond simple tissue repair. They release a range of bioactive molecules that support tissue remodeling and recovery, making them effective in treating a wide array of conditions, from cardiovascular and neurological diseases to soft tissue injuries​. Their occurrence in various tissues such as bone marrow, adipose tissue, and perinatal tissues, coupled with advances in their expansion and biobanking, ensures their accessibility and practical application in clinical settings. MSCs isolated from several tissues like bone marrow, umbilical cord, etc. have been used in clinical trials for the treatment of various diseases [3–5]. However, aging-mediated changes in the differentiation capability of MSCs pose a major setback in their use for reparative purposes [5]. Aged MSCs have a reduced capacity to differentiate into osteoblasts and chondrocytes, leading to a decreased ability to generate new bone and cartilage and repair damaged joint tissues [6].

CD45 is a protein tyrosine phosphatase and a well-known pan hematopoietic cell surface marker that is vital for their functions [7]. It is considered a negative marker for MSCs [8]. In our previous work, we demonstrated that MSCs acquire the expression of CD45 due to aging-mediated oxidative stress and activation of NF-κB signaling [9]. Doing double immunofluorescence studies using MSC-specific markers, namely CD44 and CD73 we confirmed that the CD45^+^ population within the aged MSCs was of purely mesenchymal origin, and was devoid of any hematopoietic cell contamination [9]. However, whether CD45 is actively involved in the altered differentiation properties of aged MSCs was not investigated. Although several molecules have been identified in regulating the osteoblastic and adipogenic differentiation profile of MSCs [10–14] to date, the role of CD45 in this regulation has not been investigated. In this work we used pharmacological inhibitors to inhibit CD45 activity in aged MSCs [15]. Here, for the first time, we showed the active involvement of CD45 in the deregulated differentiation potential of aged MSCs and also rejuvenating ability of pharmacological inhibitor of CD45 protein tyrosine phosphatase activity has been deciphered.

We investigated the molecular mechanism involved in the differentiation defects in the aged MSCs and found that aged MSCs display a dephosphorylated status of several regulatory kinases such as p38, p44/42, Src, and Gsk3β kinases, and pharmacological inhibition of CD45-specific protein tyrosine phosphatase (PTP) activity restores their phosphorylated status. Importantly, pharmacological inhibition of CD45 PTP activity in the aged MSCs leads to the restoration of their differentiation-specific gene profile to young-like, clearly indicating the active role of CD45 in the deregulation of differentiation in aged MSCs. Consistent with these data, young MSCs independently treated with inhibitors of these kinases acquire aging-like gene signatures. These data demonstrate that p44/42, p38, Gsk3β, and Src kinase pathways are regulated by CD45 phosphatase activity, leading to the alteration in the differentiation potential of MSCs. Importantly, we also show that consistent with the expression of osteoclastic markers in the aged MSCs [9], their secretome affects the viability and differentiation potential of primary chondrocytes, and this detrimental effect is reversed by the treatment of aged MSCs with CD45-specific PTP inhibitor.

In summary, we demonstrate that aging-mediated expression of CD45 in the MSCs affects their differentiation potential by dephosphorylation of several regulatory protein kinases. Our data indicate that CD45 can be considered a therapeutic target to rejuvenate aged MSCs for their use in regenerative medicine applications (Graphical Abstract). Ours is perhaps the first report showing the importance of CD45 in the biology of MSCs.

## Materials and methods

### Animals

Young (6–8 weeks old) and aged (20 months old) C57BL/6 female mice were housed in the institutional experimental animal facility of Symbiosis School of Biological Sciences (SSBS), Pune. All procedures were performed in compliance with relevant laws and institutional guidelines and have been approved by the Institutional Animal Ethics Committee (IAEC). IAEC approval no. SSBS/IAEC/05-2020.

6–8 weeks old male Wistar Rats weighing 120–150 gm were procured from Global BioResearch Solutions Pvt. Ltd. Pune. (CPCSEA Registration Number: 1899/PO/Bt/S/16/CPCSEA), and housed in the institutional animal facility. IAEC approval No. SSBS/IAEC/03-2022.

### Isolation and culture of MSCs

Bone marrow (BM) cells were harvested from the femur and tibia of young (6–8 weeks; young MSCs) and aged (20 months old; aged MSCs) C57BL/6 female mice. The mononuclear cells were seeded in IMDM (HiMedia, Mumbai, India) supplemented with 10% FBS (Gibco, USA) and incubated at 37 °C in 5% CO_2_. The medium was replaced every 72 h. The adherent MSCs were passaged after 60–70% confluence [16]. The cells in the third passage were used in the experiments.

### Pharmacological inhibition of tyrosine phosphatase activity of CD45

Aged MSCs were treated with 3 µM concentration of CD45 specific PTP activity inhibitor [N-(9,10-Dioxo-9,10-dihydro-phenanthren-2-yl)-2,2-dimethyl-propionamide] (Sigma Aldrich (USA) Cat. no. 540215) for 48 h (as per the data sheet and previously published paper) [15]. The aged MSCs treated with equivalent amounts of DMSO were kept as vehicle control. The inhibitor/DMSO was replaced after every 24 h.

### Osteogenic, adipogenic, and chondrogenic differentiation of aged MSCs after CD45 PTP inhibitor treatment

Briefly, to assess the effect of CD45-PTP inhibitor on aged MSCs’ differentiation potential, they were pre-treated with the PTP inhibitor for 48 h with media replacement every 24 h as described above. After 48 h, the PTP inhibitor-containing media was replaced by the specific differentiation media. The young, aged, and PTP inhibitor-treated aged MSCs were cultured in adipogenic (StemPro, Thermo Fisher, USA) (Cat no. A1007001) osteogenic (StemPro, Thermo Fisher, USA) (Cat no. A1007201), and chondrogenic (StemPro, Thermo Fisher, USA) (Cat no. A1007101) differentiation media separately for 3–4 weeks with media replenishment every 72 h.

MSCs cultured in adipogenic differentiation medium for 3 weeks were stained with Oil red O (Sigma Aldrich, USA Cat. no. 1024190250) and observed under a phase contrast microscope (Carl ZEISS, Germany) [17]. For quantitative analysis, the dye was eluted by using 2-propanol, and the absorbance of the eluates was determined at 540 nm using the Synergy H1 microplate reader (Biotek, USA).

MSCs cultured in the osteogenic medium for 21 days were stained with Alizarin red dye (Sigma Aldrich, Cat no. 1062780025) and observed under a phase contrast microscope (Carl ZEISS, Germany) [18, 19]. For quantification, 1 mL of 10% Cetylpyridinium chloride (Sigma Aldrich, Cat no. C1000000) in 10 mM sodium phosphate buffer (pH 7) was added to the cells and incubated for 60 min for dye elution, and absorbance of the eluates was determined at 550 nm using Synergy H1 microplate reader (Biotek, USA).

For chondrogenic differentiation, the cells were cultured in chondrogenic media for 21 days. After incubation, the cells were stained with Safranin O (Sigma Aldrich, USA, Cat no S8884) and observed under a phase contrast microscope (Carl Zeiss, Germany). The number of Safranin^+^ patches per field was scored.

For the alkaline phosphatase (ALP) activity assay, the cells were collected by trypsinization and the cell pellets from three separate groups (young, aged, and PTP inhibitor-treated aged MSCs) were taken in three different microcentrifuge tubes, and suspended in 300 μL of passive lysis buffer (Promega, Madison, WI, USA). Then the samples were lysed by using a probe sonicator (Sonics, Vibra Cell, USA). The samples were sonicated at 40% power for 30s X 6 times. Then the lysates were centrifuged at 10,000 rpm for 5 min, supernatants were collected and assessed for DNA content using the Quant-iT PicoGreen double-stranded DNA kit (Invitrogen, USA). The intracellular ALP activity was measured by incubation of supernatants with an alkaline phosphatase substrate, para-nitrophenyl phosphate (Sigma Aldrich, Cat no. P4744) at 37°C for 30 min. After incubation, readings were taken at 405 nm by using a plate reader (BioTeK Microplate Reader H1M GENS V3.03, USA). The ALP activity for each sample was determined by normalizing with the DNA content and was recorded as units per mg of DNA.

### Reactive oxygen species (ROS)

The young, aged, and PTP inhibitor-treated aged MSCs were trypsinized and collected in microcentrifuge tubes. The ROS levels in the cells were assessed by adding DCFHDA dye (Sigma Aldrich, USA. Cat no. D6883-50MG) (5 μM) and incubating them in the dark at RT for 15 min [9]. The percentage of DCF^+^ cells and their mean fluorescence intensity (MFI) were recorded using a flow cytometer (FACS Melody; BD Biosciences). The data were analyzed using FlowJo software (Version 7, NJ, USA). Expression of oxidative stress-specific genes was quantified by performing qRT-PCR.

### Effect of CD45-specific PTP inhibitor on NF-κB signaling

Young, aged, and PTP inhibitor-treated aged MSCs were stained with an anti-NF-κB antibody raised in rabbit (Cell Signaling Tech, USA Cat no. #8242). This antibody recognizes endogenous levels of total NF-κB p65/RelA protein. It does not cross-react with other NF-κB/Rel family members. The cells were washed and then incubated with anti-rabbit IgG (H + L), F(ab′)2 Fragment (PE Conjugate) (Cell Signaling technologies, Cat no.79408). The nuclei of the cells were stained with DAPI (Sigma Aldrich). The slides were mounted and visualized on ApoTome.2 fluorescence microscope (Carl ZEISS, Germany). Ten cells each from five non-overlapping fields were imaged and analyzed for MFI with the help of image J software (U.S. National Institutes of Health, Bethesda, Maryland, USA). For statistical analysis, Two-way ANOVA was used followed by the Bonferroni post hoc test in GraphPad Prism version 6.0 (GraphPad Software Inc, La Jolla, CA). The activation of NF-κB in both groups was measured by analyzing the nucleus: cytoplasm ratio of the NF-κβ signal.

### Immunofluorescence staining

Young, aged, and PTP-inhibitor-pre-treated aged MSCs were cultured in osteogenic and adipogenic medium for 3–4 weeks. Then the cells were washed with 1X PBS, fixed in 4% paraformaldehyde (Sigma Aldrich), and permeabilized using 0.3% Triton X-100 (Sigma Aldrich) for 15–20 min at room temperature. The cells were blocked with 3% BSA (HiMedia laboratories, India) in 1× PBS for 1 h at room temperature and then incubated with rabbit anti-RUNX2 (Cat no. 8486), -PPARG (Cat no. 2435) and -NF-κβ (Cat no. 8242) antibodies (Cell Signaling Technologies, MA, USA) overnight at 4 °C. After incubation, the cells were washed and incubated with anti-rabbit IgG (H + L), F(ab′)2 Fragment (Alexa Fluor^®^ 488 Conjugate) (Cell Signaling Technologies, Cat no. 4412), and anti-rabbit IgG (H + L), F(ab′)2 Fragment (PE Conjugate) separately (Cell Signaling Technologies, Cat no. 79408). The nuclei of the cells were stained with DAPI (Sigma Aldrich, USA Cat no. #4083). The slides were mounted and visualized on ApoTome.2 fluorescent microscopes (Carl ZEISS, Germany).

The positively stained cells were quantified by counting at least 10 cells from five non-overlapping fields and analyzed for MFI with the help of Image J software (U.S. National Institutes of Health, Bethesda, Maryland, USA). For statistical analysis, Two-way ANOVA was used, followed by the Bonferroni post hoc test in GraphPad Prism version 6.0 (GraphPad Software Inc, La Jolla, CA).

### Total RNA isolation and quantitative RT-PCR

Total RNA was extracted from young, aged, and PTP inhibitor-treated aged MSCs using the Tri reagent (Sigma Aldrich, USA, Cat no. 93289). The quantity and purity of RNA were checked on the Synergy H1 microplate reader (Biotek, USA). 1 μg of total RNA from each sample was used for reverse transcription by using the Primescript 1st strand cDNA synthesis kit (Takara, Japan). QuantStudio 3 Real-Time PCR machine (Thermo Fisher Scientific) was used for Quantitative RT-PCR studies by using SYBR Green enzyme (Thermo Fisher Scientific). The threshold values (Ct) of each sample were normalized to its *GAPDH* (housekeeping gene) ct value. The rationale behind the choice of the genes assessed and the primer sequences used for qRT-PCR have been given in Table 3. The fold change in expression was calculated by the 2^− ΔΔCt^ method with respect to young MSCs (Considered as 1).

### Western blot

To determine the molecular pathways regulated by CD45 in the aged MSCs, we examined the phosphorylation status of kinases such as p38, MAPK, SAPK/JNK, GSK3β, and Src kinase which are known to have a role in MSCs differentiation. Young, aged, and PTP inhibitor-treated aged cells were lysed and protein concentrations were determined by a micro-BCA protein estimation kit (Invitrogen. Cat no. A55860). The proteins were separated in SDS polyacrylamide gels and the separated proteins were electrically transferred to the PVDF membrane. The membranes were blocked using 5% BSA in TBST (pH − 7.6) for 2 h and then incubated overnight with primary antibodies (listed in Table 2). The membranes were thoroughly washed and incubated with appropriate HRP-conjugated secondary antibodies (Table No. 2). The signals were detected using LumiGLO (Cell Signaling, Cat no. 7003) substrate using Chemi Doc (Bio-Rad). Densitometric analysis of the protein bands was determined using Image J software. The band intensities of phosphorylated proteins were normalized with respect to that of their native (nonphosphorylated) forms, and the ratio of phosphorylated proteins versus their respective native proteins was plotted. Equal loading of phospho and native proteins was checked by comparing their signal with that of β-Actin (housekeeping protein). The membranes were also probed with an antibody to acid phosphatase 5 (raised in rabbit, Invitrogen, USA) and an anti-rabbit -HRP-tagged antibody. The signal was normalized with that of β-Actin protein (housekeeping protein).

### Pharmacological inhibition of p38, ERK1/2, SAPK, and Src signaling pathways in young MSCs

Young MSCs were treated with p38 MAPK inhibitor (PD169316, 10 μM), MEK/ERK inhibitor (PD98059, 5 μM), Src inhibitor (PP1, 10 μM), a combination of p38 inhibitor (PD169316, 5 µM), and MEK/ERK inhibitor (PD98059, 2.5 µM) for 48 h, separately [17, 20, 21] (Table No. 4). The young MSCs treated with equivalent concentrations of DMSO (Sigma Aldrich, USA. Cat. no. D2650) were used as vehicle control. The cells were lysed and subjected to qRT-PCR analysis.

### Effect of MSC secretome on cultured rat Chondrocytes

Rat chondrocytes were isolated from the femoral condyle region and were digested using Collagenase II (1 mg/mL) (Gibco, Cat no. 17101015) for 3 h. These cells were incubated in DMEM (Gibco, Cat no. 10938025) without glutamine, and supplemented with 10% FBS and 50 µg/mL 2-Phospho-l-Ascorbic acid trisodium salt. This medium is termed the chondrogenic medium in this text [22].

Conditioned medium was collected from young MSCs (YCM), aged MSCs (ACM), and aged MSCs pre-treated with CD45-specific PTP-inhibitor (PTP-ACM) cultured in 2% FBS-supplemented chondrogenic medium for 48 h, and the spent medium was centrifuged at 2600*g* for 10 min at 4 °C. The supernatant was collected and stored at − 80°C till used. Chondrocytes were treated with CM preparations, and after 48 h of the incubation period, the cells were lysed. Chondrocytes cultured in 2% FBS supplemented chondrocyte medium served as control. The lysates were used to prepare cDNA and chondrocyte-specific genes like *Collagen 2A* and *SOX9* were studied through qRT-PCR.

The viability and cytotoxicity of the chondrocytes after the treatment with different CM preparations were assessed by using the LIVE/DEAD^TM^ kit (Invitrogen, USA. Cat no. L3224). Briefly, 2 × 10^4^ chondrocytes were seeded on coverslips and treated with YCM, ACM, and PTP-ACM separately for 48 h. After 48 h, conditioned media were removed and the cells were incubated with 500 µL of the dye reagents (4 mM Calcein AM and 2 mM Ethidium-homodimer-1) for 15 min at 37 °C. Then the cells were washed with PBS to reduce non-specific binding and were observed using a fluorescence microscope (Carl Zeiss Microscope Apotome HXP 120, Germany) at excitation and emission wavelengths of 528 nm and 617 nm, respectively. The viability count for live (green) and dead (red) cells was recorded in each image manually (at least 5 images/group) using ImageJ software and % viability was calculated [23] (Additional File. 2).

### Statistical analysis

All data are represented as mean ± SD. All the experiments were done in triplicate. In the immunofluorescence study, for comparison between the two groups, an unpaired t-test was conducted. P value < 0.05 was considered statistically significant. GraphPad Prism software (version 8, San Diego, CA) was used for the statistical analysis. Statistical significance is indicated by *, where *p < 0.05; **p < 0.01; ***p < 0.001; ****p < 0.0001.

For gene expression studies between different groups, Data represent the relative expression of transcripts normalized to that of GAPDH and expressed as the Mean ± SEM for three biologically independent experiments (N = 3). For statistical analysis, one-way ANOVA followed by Bonferroni post hoc test was conducted in Graph pad prism 6 version 6.0 (GraphPad Software Inc, La Jolla, CA). Error bars in the graph indicate the standard deviation (SD). Statistical significance is indicated by *, where *p < 0.05; **p < 0.01; ***p < 0.001; ****p < 0.0001 as compared to young MSCs. ns denotes Non-significant.

Details of antibodies, inhibitors, and primer sequences are given in the Supplementary Data Table 1–4.

## Results

### Pharmacological inhibition of CD45 tyrosine phosphatase activity in the aged MSCs improves their differentiation profile

Our previous study showed that the upregulated expression of CD45 is associated with differentiation defects in the aged MSCs [9]. In this present study, we investigated whether CD45 plays an active role in the differentiation defects of aged MSCs.

First, we used different concentrations of CD45 protein tyrosine phosphatase (PTP) activity inhibitor to treat aged MSCs as per the data sheet and previously published paper [15] to determine a non-toxic concentration of the inhibitor.

We found that aged MSCs treated with 3 µM concentration of CD45- specific PTP inhibitor for 48 h was non-toxic to the MSCs as determined by the retention of their morphological characteristics and viability (Additional File. 1). At this concentration, PTP did not affect the growth rate of the aged MSCs (data not shown). Hence, 3 µM of PTP inhibitor was used in the subsequent experiments and the young MSCs treated with equivalent amounts of DMSO were kept as vehicle control.

**Fig. 1:**
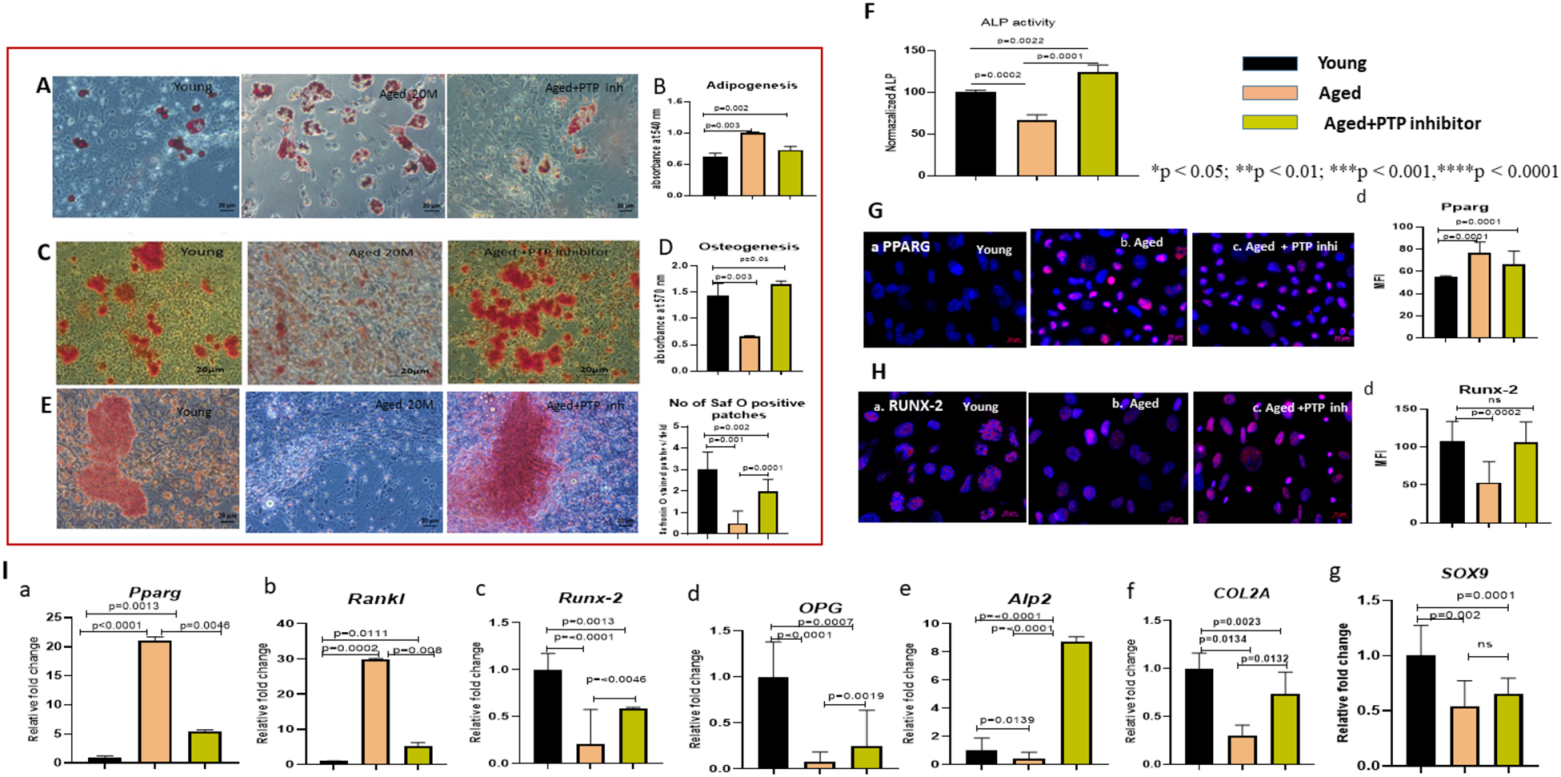
Pharmacological inhibition of CD45-specific phosphatase activity restores the differentiation potential of aged MSCs. **A** Adipogenic differentiation of young, aged, and PTP inhibitor-treated aged MSCs after Oil Red O staining is depicted. **B** Absorbance of Oil red O dye eluates from young, aged, and PTP inhibitor-treated aged MSCs in comparison to young counterpart measured at 540 nm is graphically represented. **C** The panel shows osteoblastic differentiation in young, aged, and PTP inhibitor-treated aged MSCs after alizarin Red staining. **D** Absorbance of Alizarin Red dye eluates from young, aged, and PTP inhibitor-treated aged MSCs in comparison to young counterpart measured at 570 nm is graphically represented. **E** Chondrogenic differentiation of young, aged, and PTP inhibitor-treated aged MSCs after Safronin O staining is depicted. Microscopic scoring of Safranin O positive patches is graphically represented on the right-hand side of **E**. **F** ALP activity normalized to the DNA content in young, aged, and PTP inhibitor-treated aged MSCs is depicted. **G** Immunofluorescence staining of young (**a**), aged (**b**), and PTP inhibitor-treated aged MSCs (**c**) with an antibody against PPARG. MFI of PPARG in the nuclear region in the aged MSCs is significantly high as compared to that in the young MSCs, whereas in the PTP inhibitor-treated aged MSCs it is significantly decreased (**G** **d**). **H** Immunofluorescence staining of young, aged, and PTP inhibitor-treated aged MSCs with an antibody against RUNX2 (**H** **a**–**c**). MFI for RUNX2 showed a significant increase in PTP inhibitor-treated aged MSCs than their aged counterparts (**H** **d**). Error bars in the graph indicate the standard deviation (SD). Statistical significance shown by *, where *p < 0.05; **p < 0.01; ***p < 0.001; ****p < 0.0001, *ns* not significant. compared to young. **I** qRT-PCR analysis for the relative expression of *Pparg* (**a**), *Rankl* (**b**), *Runx-2* (**c**), *OPG *(**d**),* Alp2* (**e**), *Col2A* (**f**), and *Sox9* (**g**) in young, aged and PTP inhibitor-treated aged MSCs, respectively. Data represent relative expression of transcripts normalized to that of *GAPDH* and expressed as the Mean ± SEM for three biologically independent experiments (n = 3). For statistical significance, one-way ANOVA followed by Bonferroni post hoc test in Graph pad prism 6 version 6.0 (GraphPad Software Inc, La Jolla, CA) was used. All the data are represented as mean ± SD. *p < 0.05; **p < 0.01; ***p < 0.001; ****p < 0.0001, *ns* not significant. *MSCs* mesenchymal stem cells, *PTP* protein tyrosine phosphatase, *MFI* mean fluorescence intensity

Young, aged, and aged MSCs after pre-treatment with PTP inhibitor for 48 h were subjected to osteogenic, adipogenic, and chondrogenic differentiation separately. We found that similar to that seen in the young MSCs, the PTP inhibitor-treated aged MSCs showed the presence of very few Oil Red O positive cells, whereas the untreated aged MSCs showed a large number of Oil Red O positive cells (Fig. 1A). Consistent with this observation, the Oil Red O dye eluates from the PTP inhibitor-treated aged MSCs gave a significantly lower absorbance than that from the untreated aged MSCs (Fig. 1B). Similarly, we also found that the PTP inhibitor-treated aged MSCs showed significantly enhanced osteogenic differentiation, which was at par with their young counterparts (Fig. 1C). Alizarin red dye elution study showed that the dye eluate from the CD45-PTP inhibitor aged MSCs gave a significantly higher absorbance, as compared to that given by the untreated aged MSCs, and it was comparable to that given by the young MSCs (Fig. 1D). Aged MSCs failed to show optimal chondrogenic differentiation (Fig. 1E, middle panel) as assessed by Safranin O staining. However, after the treatment with the CD45-specific PTP inhibitor, they efficiently underwent chondrogenic differentiation as indicated by a strong reactivity with the Safranin O dye (Fig. 1E right-hand panel). Scoring for safranin O-positive patches showed that the number of patches formed by PTP inhibitor-treated aged MSCs was significantly higher than the untreated aged MSC, though lower than those formed by the young MSCs (Fig. 1E).

### CD45-specific PTP inhibitor evokes differentiation-specific genes and proteins in the aged MSCs

In this set of experiments, we determined whether the changes in the differentiation profile of aged MSCs happen at gene and protein levels immediately after 48 h of treatment with the PTP inhibitor.

The ALP activity of young, aged, and aged MSCs treated with the PTP inhibitor for 48 h was determined by using an ALP activity assay kit, and the readings were normalized to the DNA content. We found that the inhibitor-treated aged MSCs showed a significant increase in alkaline phosphatase activity that was even higher than that of the young MSCs (Fig. 1F, 1st and 3rd bars). Immunostaining of these cells with antibodies against PPARG and RUNX-2 also showed the rejuvenating effect of the PTP inhibitor on the aged MSCs as evidenced by the significantly decreased expression of PPARG (Fig. 1G a–d) coupled with a significantly increased expression of RUNX-2 (Fig. 1H a–d) in them.

Gene expression studies showed a significant down-regulation of adipocyte marker *Pparg* (Fig. 1I a) and Osteoclastogenic marker *Rankl* (Fig. 1I b), and a significant up-regulation of osteogenic markers *Runx-2* (Fig. 1I c)*, OPG* (Fig. 1I d), and *Alp2 *(Fig. 1I e) in the PTP inhibitor-treated aged MSCs, as compared to their untreated aged counterparts. Interestingly, the expression of *Alp2* in PTP inhibitor-treated aged MSCs was significantly higher than that in young MSCs (1I e). PTP-inhibitor-treated aged MSCs showed a significant upregulation of chondrogenic marker *Col2A* compared to their untreated aged counterpart, but PTP inhibitor did not affect the *SOX9* expression (Fig. 1I f, g).

Collectively, these data show that pharmacological inhibition of CD45 tyrosine phosphatase activity restores the differentiation profile of the aged MSCs to the young-like by inducing differentiation-specific gene and protein expression in them, thereby clearly establishing the role of CD45 in the differentiation defects seen in the aged MSCs.

### CD45-specific PTP inhibitor reduces the oxidative stress in the aged MSCs

**Fig. 2:**
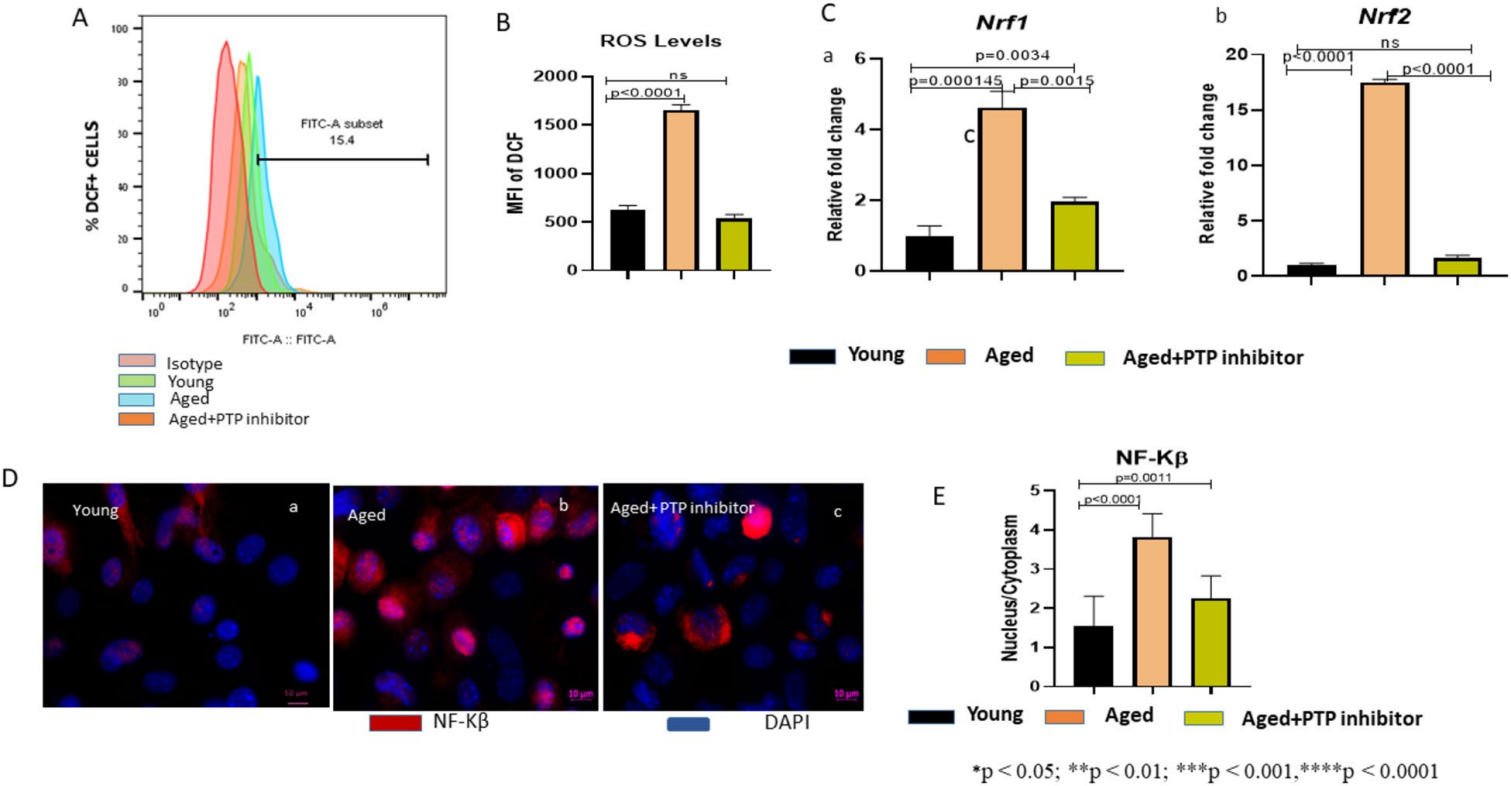
CD45-specific PTP inhibitor relieves the oxidative stress in aged MSCs. **A** Representative flow cytometry panel depicts the percentage of DCF + cells in young, aged, and aged MSCs after PTP inhibitor treatment. **B** Graphical representation shows mean fluorescence intensity (MFI) of DCF + cells in young, aged, and PTP inhibitor-treated aged MSCs. Data show a significant reduction in ROS levels in the aged MSCs after PTP inhibitor treatment. **C** (**C** **a**–**b**) qRT-PCR analyses for Nrf1- and Nrf2-specific mRNAs in young, aged, and PTP inhibitor-treated aged MSCs. Data represent the relative expression of transcripts normalized relative to GAPDH and expressed as the Mean ± SEM for three biologically independent experiments (n = 3). One-way ANOVA followed by Bonferroni post hoc test in Graph pad prism 6 version 6.0 (GraphPad Software Inc, La Jolla, CA) was used. All the data are represented as mean ± SD. *p < 0.05; **p < 0.01; ***p < 0.001, ****p < 0.0001; *ns* not significant. **D** Immunofluorescence staining of young (**a**), aged MSCs (**b**), and PTP inhibitor-treated aged MSCs (**c**) with an antibody against NF-κβ protein. **E** MFI of NF-kB signal was measured in 10 cells. Nuclear and cytoplasmic signals were calculated from at least 5 non-overlapping fields for each group using ImageJ software (n = 7). Nuclei vs. cytoplasm ratio of NF-κB signals is graphically represented. Scale bar 10 μm (×63 magnification). All the data are represented as mean ± SD. *p < 0.05; **p < 0.01; ***p < 0.001, ****p < 0.0001; *ns* not significant. *MSCs* mesenchymal stem cells, *PTP* protein tyrosine phosphatase, *MFI* mean fluorescence intensity

In our previous study, we showed that the increased CD45 expression is associated with high oxidative stress in aged MSCs [9]. Here we examined the effect of a CD45-specific PTP inhibitor on the ROS levels in them. We found that the PTP inhibitor treatment of the aged MSCs significantly downregulated the ROS levels in them, and this was similar to that in their young counterparts (Fig. 2A, B). Significant reduction in *Nrf1* and *Nrf2* gene expressions at mRNA level was also observed in the aged MSCs after CD45-specific PTP inhibitor treatment (Fig. 2C, a–b). *Nrf-1* and *Nrf-2* are two transcription factors that get upregulated in response to oxidative stress and their deficiency causes severe oxidative stress in the cells [24]. Aged cells show an increased expression of NF-kB indicating that they have higher ROS levels and consequently, they show higher expression of *Nrf-1* and *Nrf-2* to combat this stress. *Nrf2* is the primary regulator of antioxidant gene expression as it protects the cells by regulating the expression of antioxidant genes. Examination of some of the *Nrf2*-responsive genes like HMOX1 and NQO1 would lend more support to our data. We propose to do these studies shortly. Nonetheless, our data show that inhibition of tyrosine phosphatase activity of CD45 in the aged MSCs reduces their oxidative stress. Nuclear localization of NF-κB is a standard marker of oxidative stress in the cells. Immuno-staining of the aged MSCs with an anti-NF-κB antibody (Cell signaling Tech, USA) showed a significantly increased nuclear localization of NF-κB protein (Fig. 2D b), while the PTP inhibitor-treated aged MSCs showed a significant decrease in the nuclear localization of NF-κB protein (Fig. 2D c) in comparison to their untreated aged counterparts (Fig. 2D b), and this decreased nuclear localization of NF-κB was comparable to their young counterparts (Fig. 2D a). The nucleus-to-cytoplasm ratio of the NF-κB signal in the PTP-treated aged MSCs was significantly reduced as compared to the untreated aged MSCs (Fig. 2E). These data show that inhibition of CD45 phosphatase activity reverses the aging-mediated NF-κB activation in the aged MSCs.

### CD45-mediated dephosphorylation of various regulatory kinases in the aged MSCs: restoration by the PTP inhibitor

**Fig. 3:**
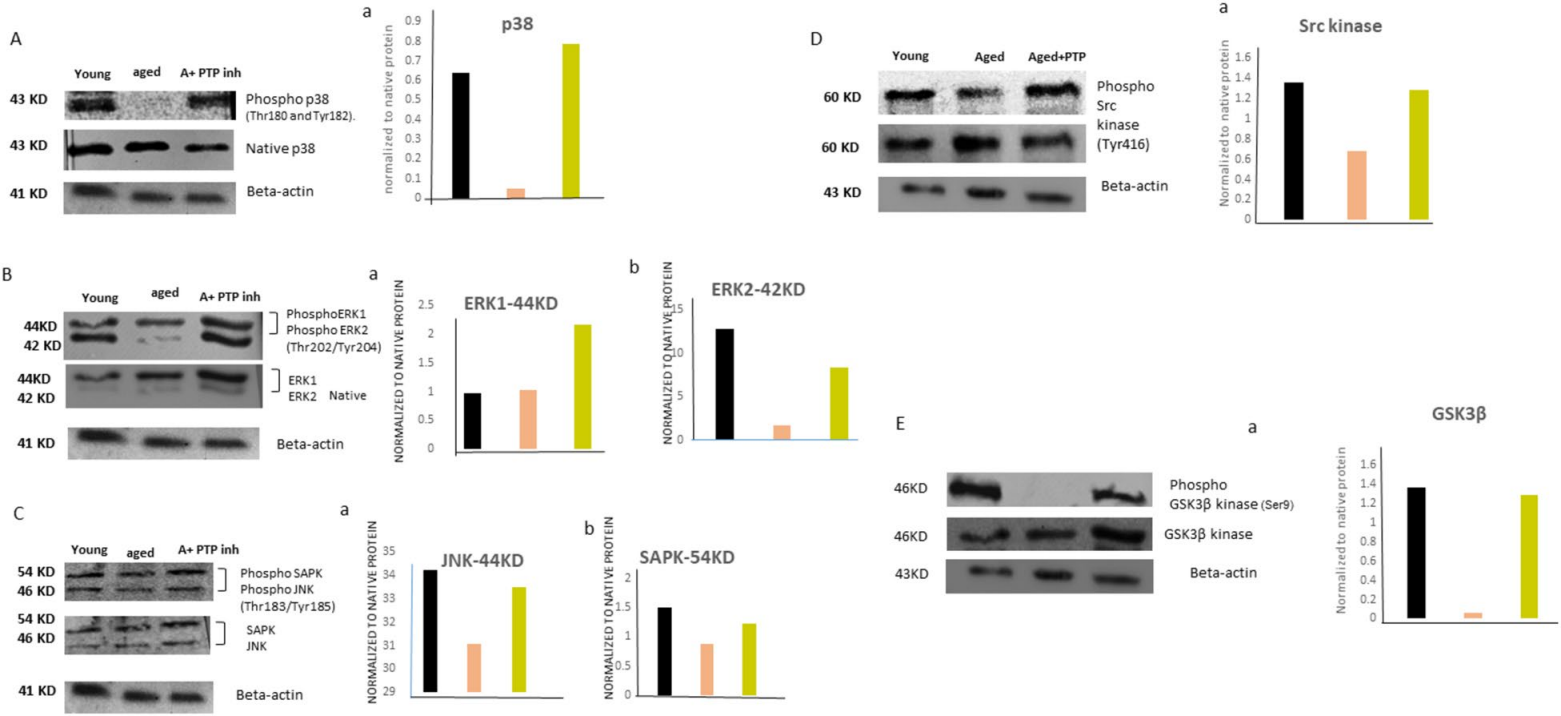
CD45-mediated dephosphorylation of various kinases in aged MSCs and its reversal by the PTP inhibitor. The figure shows the expression of phosphorylated and native forms of p38 (**A**), ERK1/2 (**B**), SAPK/JNK (**C**), Src kinase (**D**), GSK3β (**E**) and Beta-actin and their corresponding densitometry graphs [Aa, B(a–b), C(a–b), Da, Ea.] in young, aged, and PTP inhibitor-treated aged MSCs. The band intensities of phosphorylated proteins were normalized with respect to their native forms, and the fold change of the ratio of phosphorylated proteins versus native proteins was plotted. *MSCs* mesenchymal stem cells, *PTP* protein tyrosine phosphatase, *Young* young MSCs, *Aged* aged MSCs, *A + PTP−* aged MSCs treated with PTP inhibitor

Since CD45 has a tyrosine phosphatase activity, we examined whether aged MSCs show a dephosphorylated status of some of the known regulatory kinases such as p38, SAPK, JNK, Src, and GSK3β, which are the significant downstream targets of osteogenesis and also involved in adipogenesis [16, 25]. We found that in the aged MSCs, the phosphorylated form of p38 was significantly less and the PTP inhibitor treatment rescued the p38 phosphorylation state, though it was not at par with that in their young counterparts (Fig. 3A, Aa). Phosphorylation of ERK1 (44 kD) was significantly affected in the aged MSCs and it was significantly upregulated after the PTP inhibitor treatment (Fig. 3B, Ba). ERK2 (42 kD) showed a decreased expression in both native and phosphorylated forms of protein in aged MSCs and PTP treatment significantly increased the levels of both of them (Fig. 3B, Bb). Phosphorylation of SAPK/JNK expression was less in the aged MSCs (3C) and PTP inhibitor treatment showed no significant recovery of this phosphorylation state (3C, Ca, C b). Tyrosine Phosphorylation of Src was affected in the aged MSCs, as compared to that in the young MSCs (3D, D a) and PTP inhibitor treatment rescued it, and it was even higher than that in their young counterparts. Phosphorylated GSK3β was nearly absent in the aged MSCs and phosphorylation of the PTP inhibitor treatment rescued it significantly (Fig. 3E, Ea).

Collectively, the data show that aging-mediated expression of CD45 in the MSCs leads to inactivation of several kinases involved in the differentiation processes, thereby introducing the loss of osteogenesis, and gain of adipogenesis and osteoclastogenesis in them. Importantly, pharmacological inhibition of its tyrosine phosphatase activity restores the phosphorylated status of these regulatory kinases.

### Pharmacological inhibition of p38, MEK, and Src kinases in young MSCs imposes an aged-like gene expression in them

One way to cross-check whether CD45-mediated dephosphorylation of various regulatory kinases affects the differentiation of MSCs is to treat young MSCs with specific pharmacological inhibitors and examine whether their differentiation profile is affected by these treatments. In this set of experiments, we examined the effect of p38-, MEK-, and Src-specific inhibitors on the differentiation-specific gene expression of young MSCs and examined whether their gene profile becomes aged MSC-like. We treated young MSCs with inhibitors of p38 (PD169316, 10 µM), MEK/ERK (PD98059, 5 μM), and Src kinase (PP1, 10 μM) for 48 h. We also used a combination of p38 (PD169316, 5 µM) + MEK (PD98059, 2.5 µM) inhibitors. The cells were lysed after 48 h of treatment and the lysates were subjected to qRT-PCR analyses.

**Fig. 4:**
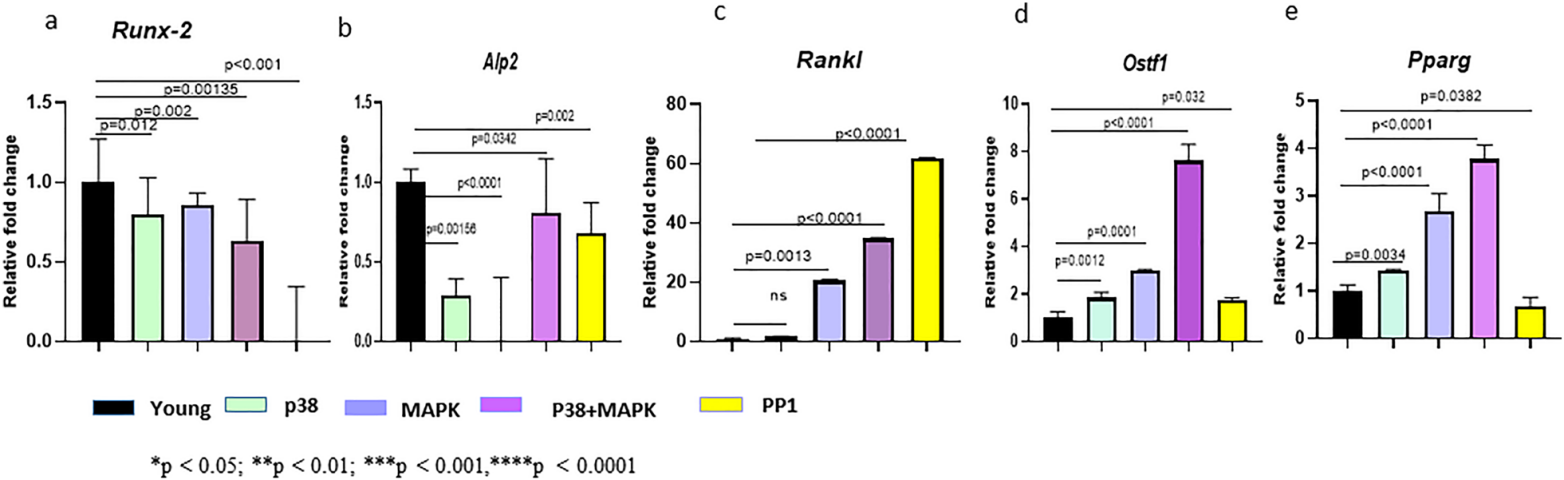
Pharmacological inhibition of p38, MAPK and SRC kinases in young MSCs imposes an aged-like gene signature on them. The figure shows qRT-PCR analyses for *Runx-2* (**a**), *Alp-2* (**b**), *Rankl* (**c**), *ostf1* (**d**), and* pparg* (**e**) gene expression at mRNA level in young MSCs treated with a p38 inhibitor, MAPK inhibitor, a combined treatment of p38 and MAPK inhibitors and PP1 separately. Data represent the relative expression of transcripts normalized relative to *GAPDH* and expressed as the Mean ± SEM for three biologically independent experiments (n = 3). For statistical analysis, between different groups, one-way ANOVA followed by Bonferroni post hoc test in Graphpad Prism 6 version 6.0 (GraphPad Software Inc, La Jolla, CA) was used. Error bars in the graph indicate the standard deviation (SD). All the data are represented as mean ± SD. *p < 0.05; **p < 0.01; ***p < 0.001, ****p < 0.0001; *ns* not significant

Results showed that inhibition of p38, MAPK, combined inhibition of p38 and MAPK, and PP1 downregulated the *Runx-2* expression in young MSCs (Fig. 4a)—*PP1 had the most striking effect* (Fig. 4a, last bar). *Alp2* expression also was significantly downregulated by all inhibitors (Fig. 4b)—the effect of MAPK inhibitor was the strongest. The combined effect of p38 and MAPK inhibitor showed down-regulation of *Alp2*, but surprisingly, it was not as effective as the treatment with individual inhibitors (Fig. 4b, 3rd bar). The effect of PP1 on *Alp2* was milder than that of p38 and MAPK inhibitors (Fig. 4b, last bar).

*Rankl* expression showed a significant upregulation after the treatment of young MSCs with MAPK, p38 + MAPK, and PP1 inhibitors (Fig. 4c), with PP1 being the most effective (Fig. 4c, last bar). Inhibition of p38 did not induce *Rankl* expression (Fig. 4c, 2nd bar). *Ostf1* expression also increased after the addition of all inhibitors (Fig. 4d), but the combined effect of p38 and MAPK inhibitors was the strongest (Fig. 4c, 4th bar). The young MSCs showed an upregulated expression of *Pparg* after treatment with all inhibitors (Fig. 4e) except PP1, indicating that perhaps Src is not involved in the adipogenic differentiation of MSCs (Fig. 4e, last bar).

Collectively, these data show that pharmacological inhibitors of p38, MEK, and Src kinases in young MSCs alter their differentiation profile and impose aged-like characteristics on them. These data also show that CD45-mediated dephosphorylation of various regulatory kinases causes differentiation defects in the aged MSCs.

### Aged MSC CM affects the viability and cartilage-specific gene expression in primary chondrocytes

In our previous study, we showed that aged MSCs express osteoclast-specific genes [9]. In the present study, we show that young MSCs treated with pharmacological inhibitors of various regulatory kinases show upregulation of osteoclast-specific gene expression (Fig. 4c, d). Hence, we examined whether this osteoclast-like gene expression in the aged MSCs also translates into their osteoclast-like detrimental effects on primary chondrocytes.

**Fig. 5:**
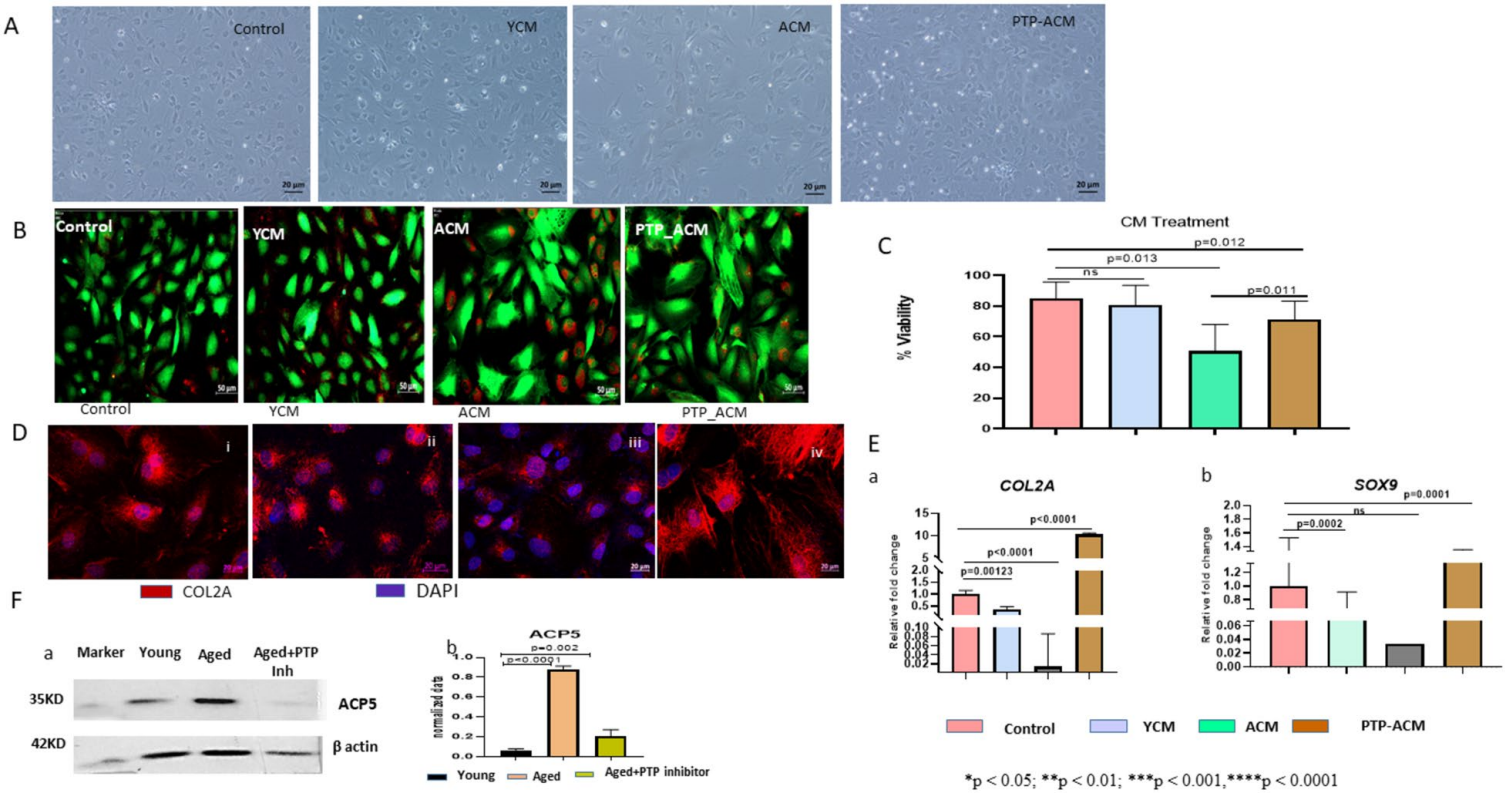
The Secretome of Aged MSCs affects the viability and differentiation of primary Chondrocytes. **A** Phase contrast images of rat chondrocytes after YCM, ACM, and PTP-ACM treatment in comparison to their control are illustrated. **B** Live/dead staining of rat chondrocytes after YCM, ACM and PTP-ACM treatment. **C** Percent viability of rat chondrocytes after YCM, ACM, and PTP-ACM treatment in comparison to control is graphically illustrated. **D** Immunofluorescence staining of control (i), YCM (ii), ACM (iii), and PTP-ACM (iv) treated chondrocytes with an antibody to Collagen 2A (COL2A). PTP-ACM (iv) treated chondrocytes showed increased expression and proper organization of Col2A. **E** qRT-PCR analysis of chondrocytes treated or not with YCM, ACM, and PTP-ACM for the relative expression of *Col2A* (**a**), *SOX9* (**b**). **F** The representative blot shows the expression of acid phosphatase type V (ACP5) their corresponding densitometry graphs [F(a, b)] in young, aged, and PTP inhibitor-treated aged MSCs. The band intensity of ACP5 was normalized with respect to housekeeping protein (Beta-actin), and the normalized values were plotted. The qRT-PCR data represent the relative expression of transcripts normalized to *GAPDH* and expressed as the Mean ± SEM for three biologically independent experiments (n = 3). For statistical analysis, One-way ANOVA followed by Bonferroni post hoc test in Graphpad Prism 6 version 6.0 (GraphPad Software Inc, La Jolla, CA) was used. All the data are represented as mean ± SD. *p < 0.05; **p < 0.01; ***p < 0.001, ****p < 0.0001; *ns* not significant. *MSCs* mesenchymal stem cells, *PTP* protein tyrosine phosphatase, *CM* conditioned medium, *YCM* young MSC-derived-CM, *ACM* aged MSC-derived CM, *PTP-ACM* PTP inhibitor treated aged MSC-derived CM

To this end, we performed a preliminary experiment to assess the effect of the secretome of the aged MSCs on the viability and differentiation of primary chondrocytes. Cartilage-derived primary chondrocytes at passage 3 were treated with YCM (young MSC CM), ACM (aged MSC CM), and PTP-ACM (CM of aged MSCs pre-treated with PTP inhibitor) separately for 48 h and observed under a phase contrast microscope. We found that the ACM-treated cells exhibited stressed morphology (Fig. 5A, 3rd panel), however, the morphology of the cells treated with PTP-ACM was comparable to that of the YCM-treated cells (Fig. 5A, 3rd and 4th panel). Consistent with these data, live/dead scoring of these cells showed significant cell death in ACM-treated chondrocytes (Fig. 5B), but the % viability of PTP-ACM-treated chondrocytes was significantly higher than the ACM-treated cell, though it was not at par with that of the controls (Fig. 5B, C).

Immunofluorescence staining of the cells using an antibody to Collagen 2a showed a significant reduction in collagen expression in the ACM-treated chondrocytes as compared to that in the control (Fig. 5D, i, and iii). Even the network-like fibers were fewer in number and significantly shorter in length, indicating that ACM affects the production and organization of collagen in the chondrocytes (Fig. 5D iii). PTP-ACM treated cells showed increased collagen protein expression in the form of organized network-like fibers (Fig. 5D iv). Consistent with these data, the qRT-PCR studies showed that the expression of *Col2A-* and *SOX9-*specific mRNAs was significantly reduced in the ACM-treated cells, whereas the PTP-ACM treated cells showed a significant recovery in the expression of these two genes (Fig. 5E a, b) suggesting that aged MSCs could disrupt normal bone development and maintenance processes, which in turn might cause bone resorption. Interestingly, the expression of Collagen2a at both mRNA and protein levels in the PTP-ACM-treated chondrocytes was significantly higher than that seen in the control and YCM-treated cells, indicating that perhaps treatment of aged MSCs with the PTP inhibitor not only abrogated the CD45 PTP activity but perhaps also stimulated the secretion of some chondrocyte-supportive factors in them. The arrangement of collage fibers was also strikingly better in the chondrocytes treated with PTP-ACM. The increase in osteoclast activity is known to be accompanied by an increase in the synthesis and secretion of acid phosphatase 5 [26]. Consistent with the detrimental data of ACM on primary chondrocytes, a western blot analysis of cell lysates showed that the aged MSCs have a significantly increased level of acid phosphatase 5 (AP) enzyme compared to young MSCs (Fig. 5F a, b). The AP expression was significantly downregulated by the treatment of aged MSCs with CD45-specific PTP (Fig. 5F a, b). However, an enzyme activity assay needs to be done to examine whether it is a tartrate-resistant AP. We plan to investigate this aspect in more detail.

Collectively, these data indicate that the conditioned medium of aged MSCs (ACM) contains detrimental factors that hamper the viability and differentiation of primary chondrocytes. Importantly, treating the aged MSCs with PTP inhibitor abrogated this activity, clearly indicating the role of CD45-phosphatase in the osteoclast-like activity of the aged MSCs. These data suggest that aged MSCs could affect the cartilage tissue and contribute to the development of skeletal disorders like osteoarthritis (OA). We plan to do in-depth studies in this regard.

## Discussion

Here, we, for the first time, show that aging-mediated increased expression of CD45 in the MSCs causes differentiation defects in them by dephosphorylating several regulatory protein kinases. Importantly, pharmacological inhibition of the phosphatase activity of CD45 in aged MSCs rejuvenates them and restores their differentiation profile to young-like. These data have important implications for regenerative medicine protocols using MSCs.

Traditionally, CD45 is considered a *negative phenotypic marker* for MSCs [8], and the presence of CD45^+^ population in MSC cultures is considered as *contamination* of the culture with hematopoietic cells. However, in our previous study, we, for the first time, demonstrated that MSCs express CD45 as a function of aging and this expression is associated with their reduced osteogenic differentiation and increased adipogenic and osteoclastogenic differentiation [9]. By performing double-immunofluorescence studies using MSC-specific markers namely, CD44 and CD73 together with CD45, we had clearly shown that the CD45^+^ population within the aged MSCs was of purely mesenchymal origin, devoid of any hematopoietic contamination [9]. However, at that time the precise role of CD45 in this deregulated differentiation process was not elucidated.

In the present study, we have used a 3 µM concentration of CD45 inhibitor (CAS 345630-40-2). Urbanek et al. demonstrated that this inhibitor effectively suppresses CD45 PTP activity at low micromolar concentrations (≤ 5 to 10 µM) with minimal effects on other phosphatases such as PTP1B and SHP-1 [27]. However, the effect of other chemical inhibitors of tyrosine phosphatases, including those having inhibitory effects on PTP1B and SHP-1, needs to be examined to have a comprehensive assessment of the specificity of the CD45 PTP inhibitor used in this study. Additionally, the specificity of this inhibitor can also be determined using various phospho-peptides. We propose to do these experiments shortly.

Given the structural conservation of the active site among various protein tyrosine phosphatases, the possibility of off-target effects must be carefully addressed. It is important to note that while the inhibitor used in the present study is designed to specifically target PTP activity of CD45, comprehensive studies on its possible off-target effects are limited. To address this issue and further support our hypothesis that the CD45-specific PTP inhibitor can rejuvenate the aged MSCs, we have initiated experiments with inducible shRNA constructs that specifically target the activity domain of CD45. We plan to transduce aged MSCs with these shRNA constructs and then subject them to various cell biological and biochemical analyses as done with the chemical inhibitor. The data obtained in these experiments would rule out the possibility of off-target effects of the chemical inhibitor used in this study and provide robust conclusions. In addition to these issues related to the specificity, the challenges involved in the future therapeutic use of this CD45-specific PTP inhibitor, such as delivery—systemic vs. topical/localized –, scalability for clinical use, etc., need to be addressed.

We show that CD45 plays an active role in the differentiation defects of aged MSCs by dephosphorylating several regulatory kinases. We show that a CD45-specific tyrosine phosphatase inhibitor rejuvenates the aged MSCs in terms of their osteogenic and adipogenic differentiation potential, and importantly, reduces the expression of osteoclast-specific mRNAs in them. Interestingly, the effect of the pre-treatment with the PTP inhibitor persisted throughout the differentiation period. This persistent effect would be advantageous in the rejuvenation of the aged MSCs before their clinical applications. However, the mechanistic aspect of this persistent effect needs to be studied, and whether this effect persists after serial passaging of the treated cells needs to be examined. In the present study, we have used bone marrow-derived MSCs. To determine whether such an effect can also be seen with MSCs isolated from other sources, we have performed experiments with L929 cells, a fibroblastic cell line derived from adipose tissue. Our preliminary results match with those obtained with bone marrow-derived MSC, indicating that this inhibitor could have broader potential (manuscript under preparation).

Collectively, these data demonstrate that the tyrosine phosphatase activity of CD45 negatively modulates the differentiation potential of MSCs and reduces their reparative potential. This is a very novel finding and to the best of our knowledge, ours is the first report showing the importance of CD45 in MSC biology!

Loss of osteogenic and chondrogenic potential and gain of adipogenic one by the aged MSCs is known [28]. Here we demonstrate that the loss of osteogenic and chondrogenic differentiation by the aged MSCs is restored by the treatment with the CD45-PTP activity inhibitor, clearly showing that the phosphatase activity of CD45 interferes with the osteogenic and chondrocytic differentiation of MSCs. Since the PTP inhibitor-treated aged MSCs exhibit higher ALP expression levels compared to young MSCs and the expression levels of COL2A and SOX9 in chondrocytes cultured with PTP + ACM are higher than those cultured with control medium or YCM, the data suggest that perhaps treating young MSCs with PTP inhibitor could boost their native differentiation ability and improve their efficacy in the regenerative medicine applications., but still, the specific components driving this effect are yet to be characterized. Proteomic or cytokine profiling of the secretome of aged MSCs before and after PTP treatment compared to that of young MSCs could pinpoint altered factors and strengthen the link between CD45 activity and MSC paracrine effects. This aspect needs to be formally investigated. In the future, testing CD45-PTP-inhibited MSCs in animal models of cartilage repair or bone regeneration would provide further evidence supporting the study's implications in regenerative medicine.

In our earlier study, we showed that oxidative stress-mediated activation of NF-κβ signaling is responsible for the induction of CD45 expression in aged MSCs [9]. In the current study, we find that inhibition of CD45 PTP activity reduces NF-κβ activation, and consequently, the ROS levels in the aged MSCs. These data suggest that perhaps there is a feedback loop operating in the system—aging-mediated oxidative stress increases CD45 expression in the MSCs and this upregulated CD45 further increases the oxidative stress in them, thereby perpetuating the stress cycle. Such chronic stress could lead to various disorders including the progression of OA and other skeletal disorders. Our data agree with a previous report by Singh and Aggarwal, where they demonstrated that in epithelial and myeloid cells, inhibitors of protein tyrosine phosphatase completely shut down the TNF-mediated activation of NF-kB in a time and dose-dependent manner [29]. Oncogenic Ras, coupled with inflammatory stimuli, has been shown to initiate a positive feedback loop involving NF-κB, thereby amplifying Ras activity to pathological levels [30, 31]. The feedback loop initiated by aging-mediated oxidative stress-induced CD45 expression and NF-kB activation in aged MSCs needs to be further investigated (Additional File.3). We are currently working towards this goal.

Most importantly, we have identified the mechanistic aspects of CD45-mediated effects on the differentiation defects of aged MSCs. We show that CD45 dephosphorylates several regulatory protein kinases like p38, MAPK, and Src in the aged MSCs, and this is perhaps the root cause of their deregulated differentiation profile. Importantly, the treatment of aged MSCs with CD45-specific PTP inhibitor reversed the phosphorylation status of all these kinases and restored the differentiation profile of the aged MSCs to young-like—an important finding in the context of their therapeutic use. These data were further supported by the finding that the treatment of young MSCs with specific kinase inhibitors imposed an aged-like deregulated differentiation profile on them. Consistent with the data obtained with aged MSCs, the young MSCs showed a significant upregulation of osteoclastic markers namely, *Rankl* and *Ostf1* after the combined treatment of p38 and MAPK inhibitors. Our data suggest that the phosphorylation profile of regulatory kinases could be used as a quality control parameter for the MSCs banked for therapeutic use.

The effect of the Src inhibitor, PP1 on *Rankl* expression was quite striking. RANKL plays a pivotal role in the differentiation of monocyte/macrophage lineage cells into osteoclasts [32, 33]. Our data show that PP1-mediated dephosphorylation of Src resulted in its activation in the young MSCs and imposed an osteoclastogenic phenotype on them by increasing the expression of *Rankl* in them. PP1 is a specific inhibitor of Src family-selective tyrosine kinases that acts as a competitive inhibitor of ATP binding [33]. However, Src is a family comprising several kinases [34], and thus, it would be interesting to identify the specific member of this kinase family responsible for the observed phenomenon. We propose to do these experiments shortly.

The possibility of MSCs going towards the osteoclastogenic pathway has never been investigated, as the classical osteoclasts have origin in monocyte/macrophage lineage cells. Here we demonstrate that the secretome of aged MSCs affects the viability and differentiation potential of primary chondrocytes, and this detrimental activity of the secretome was significantly reduced when the aged MSCs were pre-treated with a CD45-PTP activity inhibitor. In our study, we have used rat chondrocytes to examine the effect of the secretome collected from mouse MSCs. Since rats and mice share a very high level (83–100%) homology at the gene level [7, 35], the effect seen on rat chondrocytes would be at par with that on the mouse chondrocytes. We also show that inhibition of CD45-PTP activity in the aged MSCs leads to a significant reduction in the expression of osteoclastic markers, namely, *Rankl *and *Ostf1* (Graphical Abstract). We also found that the aged MSCs have a significantly increase expression of acid phosphatase 5, which was significantly downregulated by their treatment with the CD45-specific PTP inhibitor (Fig. 5F a, b). These findings could have important implications in the development and management of skeletal disorders like osteoarthritis (OA). However, this aspect needs to be examined in an OA model.

Our data give a clinically important meaning to the phenotypic characterization of MSCs and suggest that the presence of the CD45^+^ population in the stromal cultures should not only be taken as the mere *presence of contaminating hematopoietic cells* but also that of the aged CD45^+^ MSCs which are likely to have poor osteoblastic and chondrocytic differentiation potential and increased adipogenic one [9]. These aged MSCs could also exert osteoclastogenic activity on cartilage. Use of such CD45^+^ MSCs can either be avoided or they can be rejuvenated using the CD45-specific PTP inhibitor before their clinical use. CD45 is already being studied as a drug target to treat myeloid leukemia [36], and hence, these clinical-grade drugs can be applied for the rejuvenation of aged MSCs. Since Src activity is essential for osteoclast function [37] and it is found to be abnormally increased in the cartilage tissue of OA patients, Src inhibitors are being investigated for the treatment of OA. Our data provide an additional scientific basis for these investigations.

In this study, we have examined the CD45 expression and its consequences in the BM-derived MSCs only. Whether MSCs isolated from other tissues collected from aged animals also show higher expression of CD45 and affect their tissue-specific regenerative capacity remains to be seen.

Overall, our study has unravelled the hitherto unknown and novel role of CD45 in aging-mediated deregulated differentiation of MSCs. These findings could have important implications in regenerative medicine protocols involving MSCs.

## Conclusion

Our data show that aging-mediated increased expression of CD45 on the MSCs leads to their deregulated differentiation due to the dephosphorylation of several regulatory kinases in them. Pharmacological inhibition of the tyrosine phosphatase activity of CD45 would rejuvenate the aged MSCs and restore their reparative activity and regenerative potential.

## Supplementary Information

Additional File 1: Effect of different concentrations of CD45-specific PTP inhibitor on the viability of Aged-MSCs. A. (a, b, c). Representative phase contrast images exhibit morphological characteristics of aged MSCs treated with 3µM and 5µM CD45-specific PTP inhibitor for 6 hrs (upper panels) 24hrs (middle panels), and 48hrs (lower panels) (10X magnification scale=20µm). B. shows percent viability of aged MSCs after treatment with DMSO,3µM, and 5µM CD45-specific PTP inhibitor for 6hrs, 24hrs, and 48hrs, respectively. For statistical analysis, One-way ANOVA followed by Bonferroni post hoc test in Graphpad Prism 6 version 6.0 (GraphPad Software Inc, La Jolla, CA) was used. All the data are represented as mean ± SD. *p < 0.05; **p < 0.01; ***p < 0.001, ****p < 0.0001; ns: not significant

Additional File 2: The secretome of aged MSCs has detrimental effects on the viability and differentiation of primary chondrocytes. Aged MSCs showed expression of osteoclast-specific markers, and hence, the effect of their secretome on primary chondrocytes was examined. Chondrocytes treated with aged MSCs’ conditioned media (ACM) exhibited stressed morphology and reduced viability. However, the CM of aged MSCs treated with PTP-inhibitor (PTP-ACM) did not have such adverse effects on the chondrocytes. As expected, the YCM-treated chondrocytes showed good viability and differentiation potential. Abbreviation for Additional File 3: Mesenchymal Stem Cells (MSCs), Young Mesenchymal Stem cells derived Conditioned Medium (YCM), Aged Mesenchymal Stem cells derived Conditioned Medium (ACM) Protein tyrosine phosphatase inhibitor treated-ACM (PTP-ACM)

Additional File 3: A feedback loop in CD45 expression and oxidative stress induction. Aging-induced oxidative stress leads to the expression of CD45 in the MSCs. The phosphatase activity of CD45 further contributes to the escalation of ROS (Reactive Oxygen Species) levels and activation of NF-κβ in them. These increased ROS levels sustain the CD45 expression in the aged MSCs, thereby forming a feedback loop. Such chronic stress could contribute to disorders like osteoarthritis (OA)

Additional file 4

Figure I, II, III depict a representative blot from three biological replicates probed with different antibodies. Figure I Figure A) full length blot of native ERK 1/2. Molecular weight ERK 1 is 44KD and ERK2 is 42KD. Figure B) full length blot of phospho ERK 1/2. Molecular weight ERK 1 is 44KD and ERK2 is 42KD. Figure C) full length blot of native p38. Molecular weight is 43KD. Figure D) full length blot of phospho p38. Molecular weight is 43KD. Figure E) full length blot of native SAPK/JNK. Molecular weight SAPK is 54KD and JNK is 46KD. Figure F) full length blot of phospho SAPK/JNK. Molecular weight SAPK is 54KD and JNK is 46KD. Young- (young MSCs), Aged – (Aged MSCs), A+PTP- (Aged MSCs treated with PTP inhibitor)

Figure II Figure G) full length blot of native GSK3β Molecular weight 46KD. Figure H) full length blot of phospho GSK3β Molecular weight 46KD. Figure I) full length blot of native Src Molecular weight 60KD. Figure J) full length blot of phospho Src Molecular weight 60KD. Young- (young MSCs), Aged – (Aged MSCs), A+PTP- (Aged MSCs treated with PTP inhibitor)

Figure III Figure J) full length blot of Beta actin weight 43KD. Young- (young MSCs), Aged – (Aged MSCs), A+PTP- (Aged MSCs treated with PTP inhibitor)

## Data Availability

Data sharing is not applicable to this article as no datasets were generated or analysed during the current study.
