# Supplementary figures and images for: Inhibition of CD45-specific phosphatase activity restores the differentiation potential of aged mesenchymal stromal cells: implications in regenerative medicine
Source: Biol Res. 2025 May 2;58:24. doi: 10.1186/s40659-025-00603-8 (PMC12046811; doi:10.1186/s40659-025-00603-8)

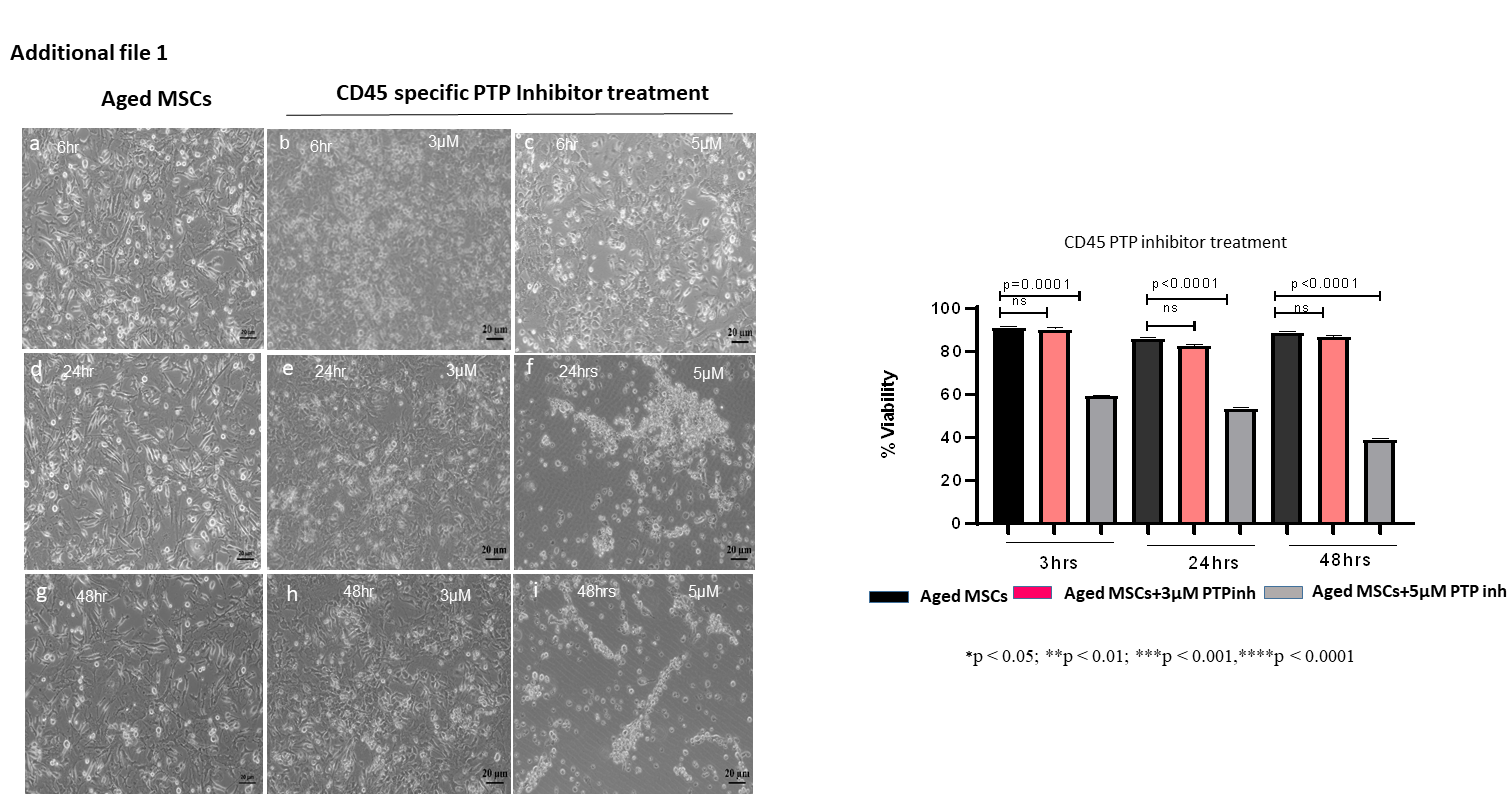

Supplement: Supplementary file 1 — Additional File 1: Effect of different concentrations of CD45-specific PTP inhibitor on the viability of Aged-MSCs. A. (a, b, c). Representative phase contrast images exhibit morphological characteristics of aged MSCs treated with 3µM and 5µM CD45-specific PTP inhibitor for 6 hrs (upper panels) 24hrs (middle panels), and 48hrs (lower panels) (10X magnification scale=20µm). B. shows percent viability of aged MSCs after treatment with DMSO,3µM, and 5µM CD45-specific PTP inhibitor for 6hrs, 24hrs, and 48hrs, respectively. For statistical analysis, One-way ANOVA followed by Bonferroni post hoc test in Graphpad Prism 6 version 6.0 (GraphPad Software Inc, La Jolla, CA) was used. All the data are represented as mean ± SD. *p < 0.05; **p < 0.01; ***p < 0.001, ****p < 0.0001; ns: not significant [file 40659_2025_603_MOESM1_ESM.tif]

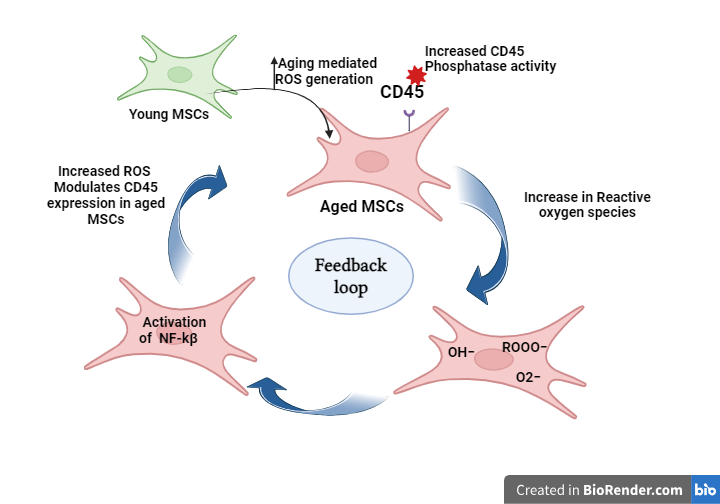

Supplement: Supplementary file 2 — Additional File 2: The secretome of aged MSCs has detrimental effects on the viability and differentiation of primary chondrocytes. Aged MSCs showed expression of osteoclast-specific markers, and hence, the effect of their secretome on primary chondrocytes was examined. Chondrocytes treated with aged MSCs’ conditioned media (ACM) exhibited stressed morphology and reduced viability. However, the CM of aged MSCs treated with PTP-inhibitor (PTP-ACM) did not have such adverse effects on the chondrocytes. As expected, the YCM-treated chondrocytes showed good viability and differentiation potential. Abbreviation for Additional File 3: Mesenchymal Stem Cells (MSCs), Young Mesenchymal Stem cells derived Conditioned Medium (YCM), Aged Mesenchymal Stem cells derived Conditioned Medium (ACM) Protein tyrosine phosphatase inhibitor treated-ACM (PTP-ACM) [file 40659_2025_603_MOESM2_ESM.tif]

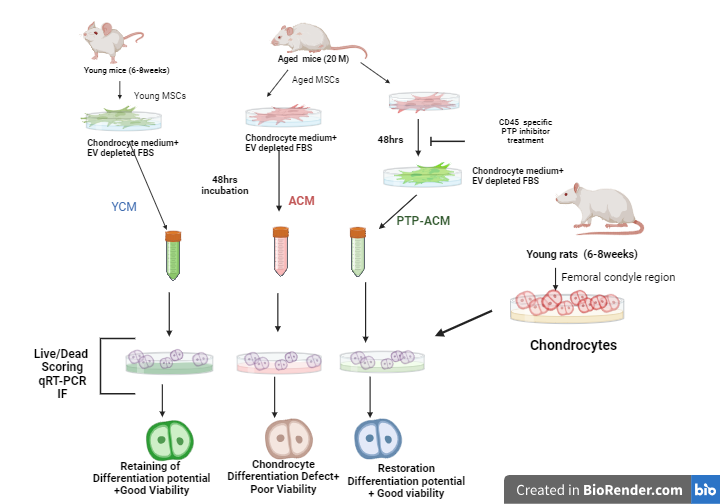

Supplement: Supplementary file 3 — Additional File 3: A feedback loop in CD45 expression and oxidative stress induction. Aging-induced oxidative stress leads to the expression of CD45 in the MSCs. The phosphatase activity of CD45 further contributes to the escalation of ROS (Reactive Oxygen Species) levels and activation of NF-κβ in them. These increased ROS levels sustain the CD45 expression in the aged MSCs, thereby forming a feedback loop. Such chronic stress could contribute to disorders like osteoarthritis (OA) [file 40659_2025_603_MOESM3_ESM.tif]

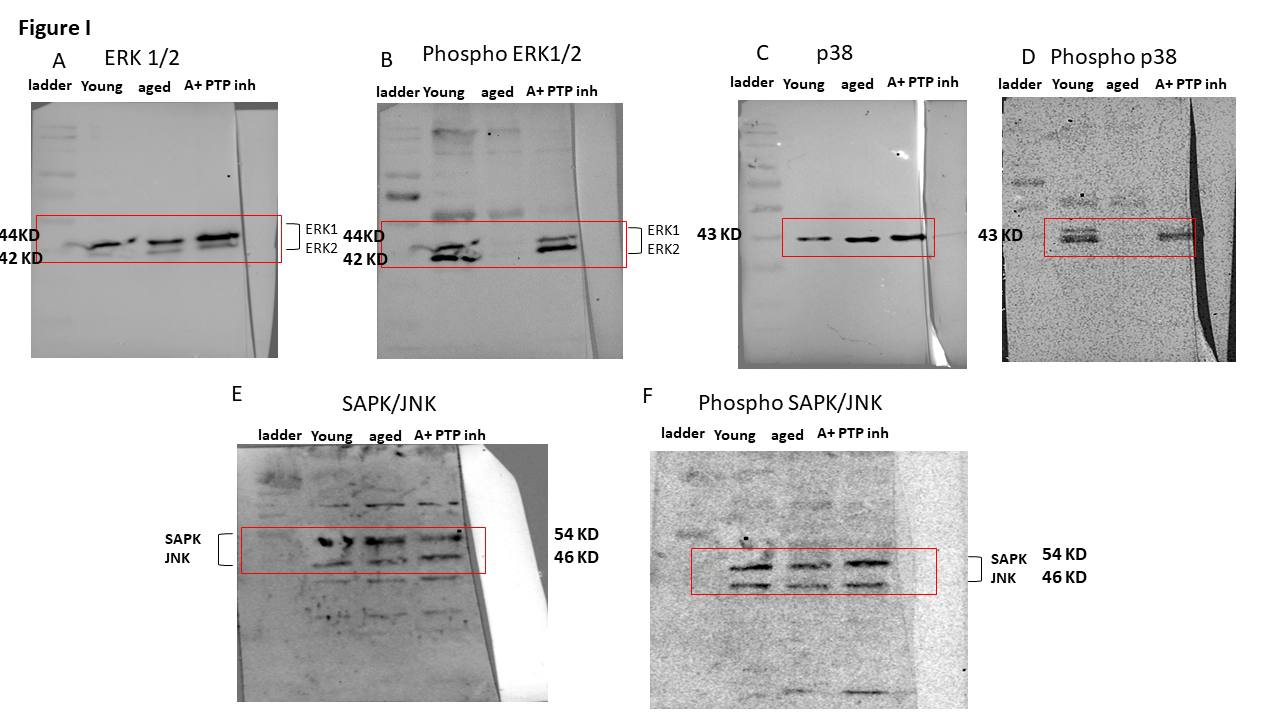

Supplement: Supplementary file 5 — Figure I, II, III depict a representative blot from three biological replicates probed with different antibodies. Figure I Figure A) full length blot of native ERK 1/2. Molecular weight ERK 1 is 44KD and ERK2 is 42KD. Figure B) full length blot of phospho ERK 1/2. Molecular weight ERK 1 is 44KD and ERK2 is 42KD. Figure C) full length blot of native p38. Molecular weight is 43KD. Figure D) full length blot of phospho p38. Molecular weight is 43KD. Figure E) full length blot of native SAPK/JNK. Molecular weight SAPK is 54KD and JNK is 46KD. Figure F) full length blot of phospho SAPK/JNK. Molecular weight SAPK is 54KD and JNK is 46KD. Young- (young MSCs), Aged – (Aged MSCs), A+PTP- (Aged MSCs treated with PTP inhibitor) [file 40659_2025_603_MOESM5_ESM.tif]

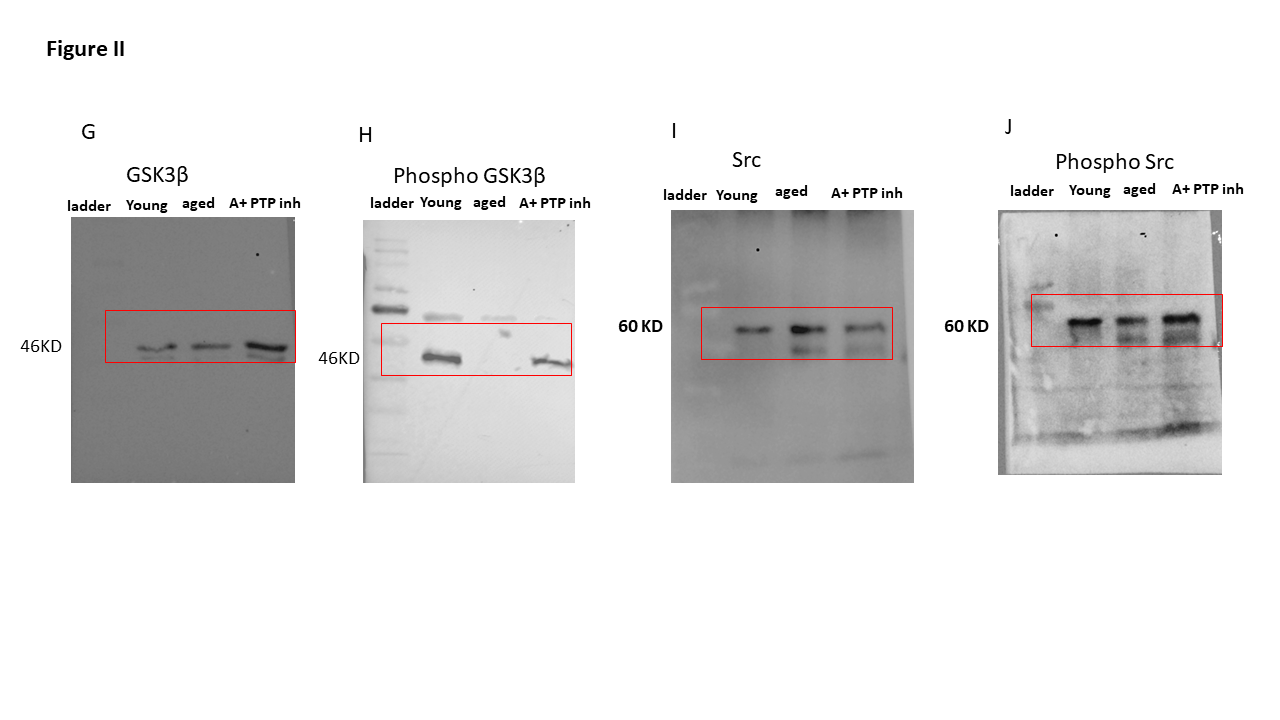

Supplement: Supplementary file 6 — Figure II Figure G) full length blot of native GSK3β Molecular weight 46KD. Figure H) full length blot of phospho GSK3β Molecular weight 46KD. Figure I) full length blot of native Src Molecular weight 60KD. Figure J) full length blot of phospho Src Molecular weight 60KD. Young- (young MSCs), Aged – (Aged MSCs), A+PTP- (Aged MSCs treated with PTP inhibitor) [file 40659_2025_603_MOESM6_ESM.tif]

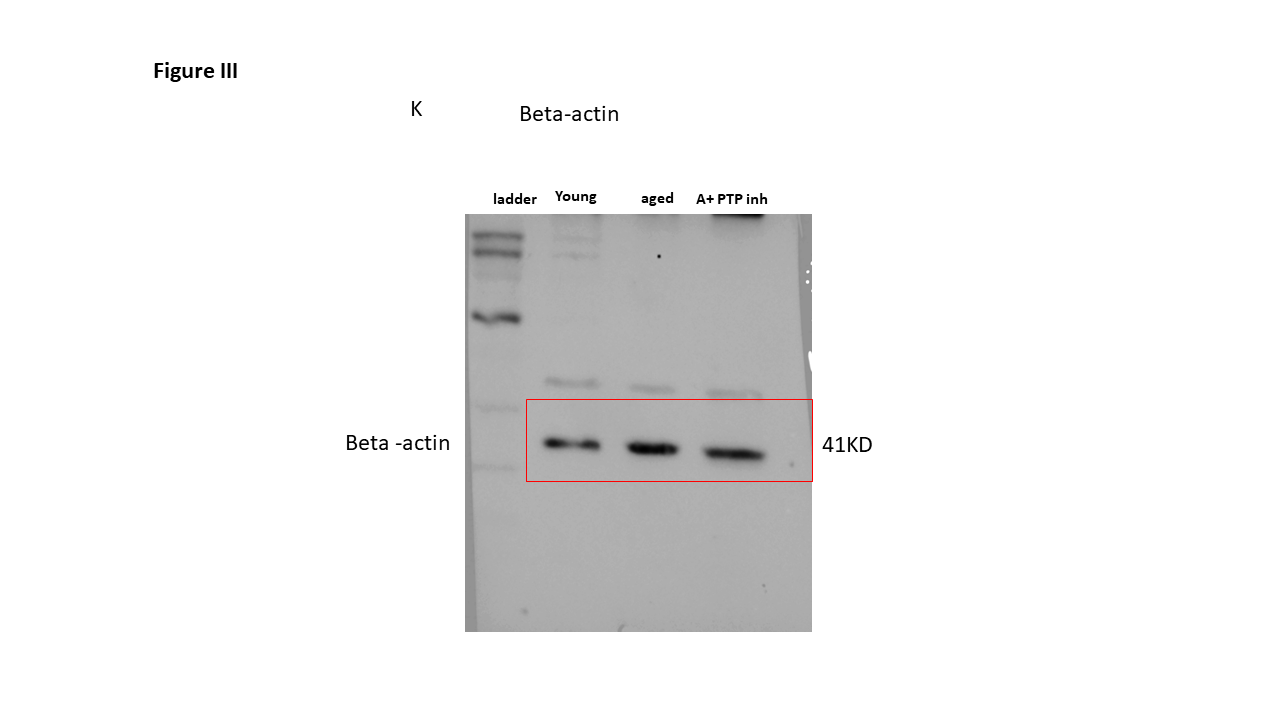

Supplement: Supplementary file 7 — Figure III Figure J) full length blot of Beta actin weight 43KD. Young- (young MSCs), Aged – (Aged MSCs), A+PTP- (Aged MSCs treated with PTP inhibitor) [file 40659_2025_603_MOESM7_ESM.tif]
