# Supplementary material for: Inhibition of CD45-specific phosphatase activity restores the differentiation potential of aged mesenchymal stromal cells: implications in regenerative medicine
Source: Biol Res. 2025 May 2;58:24. doi: 10.1186/s40659-025-00603-8 (PMC12046811; doi:10.1186/s40659-025-00603-8)
Supplement: Supplementary file 4 — Additional file 4 [file 40659_2025_603_MOESM4_ESM.docx]

**Table 1: Abbreviations**

| Sr no | Abbreviations | Full forms |
| --- | --- | --- |
|  | IMDM | Iscove's Modified Dulbecco's Medium |
|  | DCHFDA | 2'-7'-Dichlorodihydrofluorescein diacetate |
|  | PTP | Protein tyrosine Phosphatase |
|  | DAPI | 4′,6-diamidino-2-phenylindole |
|  | BM | Bone marrow |
|  | BM-MSCs | Bone marrow-derived mesenchymal stromal cells |
|  | BSA | Bovine Serum Albumin |
|  | FBS | Fetal Bovine Serum |
|  | FITC | Fluorescein Isothiocyanate |
|  | hr | Hour |
|  | IAEC | Institutional Animal Ethics Committee |
|  | IBMX | 3-isobutyl-1-methylxanthine |
|  | JNK | c-Jun N-terminal kinase |
|  | MEM | Minimal essential medium |
|  | MFI | Mean fluorescence intensity |
|  | Min | Minute |
|  | mL | Millilitre |
|  | mm | Millimetre |
|  | mM | Millimolar |
|  | μL | Microlitre |
|  | μg | Microgram |
|  | μM | Micromolar |
|  | MSCs | Mesenchymal Stromal Cells |
|  | NF-κB | Nuclear factor Kappa B |
|  | ng | Nanogram |
|  | PFA | Paraformaldehyde |
|  | PBS | Phosphate Buffer Saline |
|  | PE | Phycoerythrin |
|  | RNA | Ribonucleic acid |
|  | RT | Room temperature |
|  | qRT-PCR | Quantitative Real-time polymerase chain reaction |
|  | RPM | Revolutions per Minute |
|  | RUNX2 | Runt-related transcription factor 2 |
|  | ALP | Alkaline phosphatase |
|  | RANK | Receptor activator of NF-κB |
|  | OSTF1 | Osteoclast stimulating factor |
|  | Nrf (1 and 2) | Nuclear factor erythroid 2-related factor 1/2 (1 and 2) |
|  | SAPK | Stress-activated protein kinase |
|  | MAPK | Mitogen-activated protein kinase |
|  | JNK | c-Jun NH_2_-terminal kinase |
|  | SFK | Src family of protein tyrosine kinase |
|  | GSK3β | Glycogen synthase kinase-3 beta |

**Table 2: List of antibodies**

| Sr no | Antibody | Concentration used | Make | Catalogue no. |
| --- | --- | --- | --- | --- |
| 1 | Rabbit anti-RUNX2 | 1 µg/ml | Cell Signalling Tech (USA) | RUNX2 (D1H7) Rabbit mAb #8486 |
| 2 | Rabbit anti-PPARγ | 1 µg/ml | Cell Signalling Tech(USA) | PPARγ (C26H12) Rabbit mAb #2435 |
| 3 | Rabbit anti-NF-κB | 1 µg/ml | Cell Signalling Tech(USA) | NF-κB p65 (D14E12) XP^®^ Rabbit mAb #8242 |
| 5 | Rabbit anti phospho p38 | 1 µg/ml | Cell signalling Tech (USA) | Phospho-p38 MAPK (Thr180/Tyr182) (D3F9) XP® Rabbit mAb #4511 |
| 6 | Rabbit anti p38 | 1 µg/ml | Cell signalling Tech (USA) | p38 MAPK (D13E1) XP^®^ Rabbit mAb #8690 |
| 7 | Rabbit anti phospho SAPK/JNK | 1 µg/ml | Cell signalling Tech (USA) | Phospho-SAPK/JNK (Thr183/Tyr185) (81E11) Rabbit mAb #4668 |
| 8 | Rabbit anti SAPK/JNK | 1 µg/ml | Cell signalling Tech (USA) | SAPK/JNK Antibody #9252 |
| 9 | Rabbit anti phospho ERK (1/2) | 1 µg/ml | Cell signalling Tech (USA) | Phospho-p44/42 MAPK (Erk1/2) (Thr202/Tyr204) (D13.14.4E) XP^®^ Rabbit mAb #4370 |
| 10 | Rabbit anti ERK (1/2) | 1 µg/ml | Cell signalling Tech (USA) | p44/42 MAPK (Erk1/2) (137F5) Rabbit mAb #4695 |
| 11 | Rabbit anti phospho GSK3β | 1 µg/ml | Cell signalling Tech (USA) | Phospho-GSK-3β (Ser9) (D85E12) XP® Rabbit mAb #5558 |
| 12 | Rabbit anti GSK3β | 1 µg/ml | Cell signalling Tech USA) | GSK-3β (D5C5Z) XP® Rabbit mAb #12456 |
| 13 | Rabbit anti phospho Src | 1 µg/ml | Cell signalling Tech (USA) | Phospho-Src Family (Tyr416) Antibody #2101 |
| 14 | Rabbit anti Src | 1 µg/ml | Cell signalling Tech (USA) | Src Antibody #2108 |
| 15 | Rabbit anti ACP5 polyclonal Antibody | 1mg/ml | Invitrogen (USA) | PA5-106914 |

**Table 3: List of primers and the rationale behind the choice of genes**

| **Genes** | **Tm values** | **Sequences** | **Species** | **The rationale behind the choice of these** | |
| --- | --- | --- | --- | --- | --- |
| *GAPDH*  (Glyceraldehyde-3-phosphate dehydrogenase) | 60 | F: GGGGTCCCAGCTTAGGTTCA  R: CCAATACGGCCAAATCCGTT | Mouse | Housekeeping gene | |
| *CD45*  (protein tyrosine phosphatase) | 55 | F: ATGGTCCTCTGAATAAAGCCCA  R: TCAGCACTATTGGTAGGCTCC | Mouse | Cellular function  (here mainly focus on its role in aging) | |
| *RUNX2*  (Runt-related transcription factor 2) | 55 | F: TGACACTGCCACCTCTGACT  R: ATGAAATGCTTGGGAACTGC | Mouse | Master regulator of osteoblast differentiation | |
| *PPARG*  ((Peroxisome Proliferator-Activated Receptor Gamma) | 60 | F: AGACAACGGACAAATCACCA  R: GAAAACTGGCACCCTTGAAAA | Mouse | Master regulator of adipocyte differentiation and lipid metabolism | |
| *OPG*  (Osteoprotegerin) | 60 | F: GCAAAAAGAGGCAACCTGAGC  R: TGCTGGTTCAGCTCCACATT | Mouse | Regulates bone density and protects bone, inhibits osteoclastogenesis | |
| *ALP*(Alkaline Phosphatase ) | 60 | F: CAGGAGAAACAGGGCCTACAG  R: AGCAAAGATCCAAGACGCCG | Mouse | Early marker of osteoblast differentiation | |
| *Rankl*  (Receptor Activator of Nuclear Factor Kappa-Β Ligand) | 55 | F: AGACAACGGACAAATCACCA  R: GAAAACTGGCACCCTTGAAAA | Mouse | key regulator for osteoclast differentiation and their function. | |
| *Nrf1*  (Nuclear Respiratory Factor 1) | 60 | F: CGTTACAGGGCGGTGAAA  R: TCCAGTAAGTGCTCCGACG | Mouse | | Oxidative stress marker |
| *Nrf2*  *(Nuclear Respiratory Factor 2)* | 60 | F: TTTCAGCAGCATCCTCTCCA  R: AGCCTTCAATAGTCCCGTCC | Mouse | | A master regulator of cellular antioxidant responses |
| *Collagen 2A* | 60 | F: GAGTGGAAGAGCGGAGACTACTG  R: GTCTCCATGTTGCAGAAGACTTTCA | RAT | Key regulator of cartilage formation | |
| *Aggrecan* | 55 | F: GACCTGTGTGAGATCGACCA  R: GTTGGTTTGGACGCCACTTC | RAT | Key component of the extracellular matrix of cartilage and important for chondrocyte differentiation. | |
| *Sox9* |  | F: TCCAGCAAGAACAAGCCACA  R: CGAAGGGTCTCTTCTCGCTC | RAT | A key transcription factor that controls the differentiation and proliferation of chondrocytes | |

**Table 4: Inhibitors and Chemicals Used**

| S. No. | Name | Cat.no. | Make |
| --- | --- | --- | --- |
| 1.  2.  3.  4.  5.  6.  7.  8  9  10.  11.  12  13.  14  15.  16  17  18 | PTP inhibitor  PD169316 (p38 inhibitor)  PD 98059 (MEK Inhibitor)  PP1 (Src kinase inhibitor)  2′,7′Dichlorodihydrofluorescein diacetate  StemPro™ Adipogenesis Differentiation Kit  StemPro™ osteogenesis Differentiation Kit  StemPro™ osteogenesis Differentiation Kit  Oil red O staining solution  Alizarin red dye  [Cetylpyridinium chloride](https://www.sigmaaldrich.com/IN/en/substance/cetylpyridiniumchloride358006004246)  Safranin O  PNPP (p-nitrophenyl phosphate)  Phosphatase substrate  Tri reagent  Micro BCA Protein Assay - Pierce BCA Protein Assay Kits  20X LumiGLO® Reagent and 20X Peroxide #  Dimethyl Sulfoxide  Collagenase II | 540215  513030-1MG  513000-5MG  P0040-5MG  D6883-50MG  A1007001  A1007201  A1007101  1024190250  1062780025  C1000000  S8884  P4744  [93289](https://www.sigmaaldrich.com/IN/en/product/sigma/93289)  A55860    7003  17101015 | Sigma-Aldrich (USA)  Sigma-Aldrich (USA)  Sigma-Aldrich (USA)  Sigma-Aldrich (USA)  Sigma-Aldrich (USA)  Themofisher (USA)  Themofisher (USA)  Themofisher (USA)  Sigma Aldrich (USA)  Sigma Aldrich (USA)  Sigma Aldrich (USA)  Sigma Aldrich (USA)  Sigma Aldrich (USA)  Sigma Aldrich (USA)  Invitrogen  Cell Signaling Technology  Sigma Aldrich (USA)  Gibco |
